# Weighted Statistical Binning: Enabling Statistically Consistent Genome-Scale Phylogenetic Analyses

**DOI:** 10.1371/journal.pone.0129183

**Published:** 2015-06-18

**Authors:** Md Shamsuzzoha Bayzid, Siavash Mirarab, Bastien Boussau, Tandy Warnow

**Affiliations:** 1 Department of Computer Science, University of Texas at Austin, Austin, Texas, USA; 2 Laboratoire de Biométrie et Biologie Évolutive, Université de Lyons, France; 3 Department of Computer Science, University of Illinois at Urbana-Champaign, Urbana, IL, USA; University of Florida, UNITED STATES

## Abstract

Because biological processes can result in different loci having different evolutionary histories, species tree estimation requires multiple loci from across multiple genomes. While many processes can result in discord between gene trees and species trees, incomplete lineage sorting (ILS), modeled by the multi-species coalescent, is considered to be a dominant cause for gene tree heterogeneity. Coalescent-based methods have been developed to estimate species trees, many of which operate by combining estimated gene trees, and so are called "summary methods". Because summary methods are generally fast (and much faster than more complicated coalescent-based methods that co-estimate gene trees and species trees), they have become very popular techniques for estimating species trees from multiple loci. However, recent studies have established that summary methods can have reduced accuracy in the presence of gene tree estimation error, and also that many biological datasets have substantial gene tree estimation error, so that summary methods may not be highly accurate in biologically realistic conditions. Mirarab et al. (Science 2014) presented the "statistical binning" technique to improve gene tree estimation in multi-locus analyses, and showed that it improved the accuracy of MP-EST, one of the most popular coalescent-based summary methods. Statistical binning, which uses a simple heuristic to evaluate "combinability" and then uses the larger sets of genes to re-calculate gene trees, has good empirical performance, but using statistical binning within a phylogenomic pipeline does not have the desirable property of being *statistically consistent*. We show that weighting the re-calculated gene trees by the bin sizes makes statistical binning statistically consistent under the multispecies coalescent, and maintains the good empirical performance. Thus, "weighted statistical binning" enables highly accurate genome-scale species tree estimation, and is also statistically consistent under the multi-species coalescent model. New data used in this study are available at DOI: http://dx.doi.org/10.6084/m9.figshare.1411146, and the software is available at https://github.com/smirarab/binning.

## Introduction

The estimation of phylogenetic trees, whether of individual loci (so called “gene trees”) or at the genome-level (species trees), is a basic step in many biological analyses [1]. However, estimating gene trees and species trees with high accuracy is difficult for many reasons, including computational issues (nearly all problems are NP-hard) and dataset issues. For example, while highly accurate gene trees can be computed for some loci, when a locus has limited *phylogenetic signal* (e.g., its sequences are too short, or it evolves too slowly), its gene tree may only be estimated with partial accuracy. Species tree estimation is also difficult, because different loci can have different phylogenetic trees, a phenomenon that occurs due to several different biological processes. In particular, many groups of species evolve with rapid speciation events, a process that is likely to produce conflict between gene trees and species trees due to *incomplete lineage sorting* (ILS) [2–5]. Furthermore, when ILS occurs, standard methods for estimating species trees, such as concatenation (which combines sequence alignments from different loci into a single “supermatrix”, and then computes a tree on the supermatrix) and consensus methods, can be statistically inconsistent [6, 7], and produce highly supported but incorrect trees [8]. Because these standard methods for estimating species trees from multiple loci can be positively misleading in the presence of gene tree heterogeneity due to ILS, statistical methods (e.g., [9–13]) have been developed to estimate the species tree assuming all gene tree heterogeneity is due to ILS and, in particular, not to poor phylogenetic signal.

Here we describe one of the recent approaches for estimating the species tree from a set of multiple sequence alignments, one for each of *p* different loci on a set *S* of *n* species. We will assume that the input sequence data are generated under a multi-step process, which we now define:

Definition 1: Under the **GTR+MSC** model, gene trees evolve within a species tree under the multi-species coalescent (MSC) model, and then sequences evolve down each gene tree under the General Time Reversible (GTR) model [14]. The different gene trees are equipped with their own GTR model parameters, and so the tree topologies, 4 × 4 substitution matrices, and gene tree branch lengths can differ between the different genes.

Thus, under the GTR+MSC model, a method for estimating the species tree will begin with the sets of sequences for the different loci, and then infer the species tree. There are many different types of methods to estimate species trees from sets of sequence alignments for multiple loci, and we will refer to all of these methods as “phylogenomic pipelines”.

Definition 2: We will say that a phylogenomic pipeline is **statistically consistent** under the GTR+MSC model if, as the number *p* of loci and the number *k* of sites in the sequence alignment for each locus both increase to infinity, then the estimated species tree converges in probability to the true species tree.

There are many phylogenomic pipelines that are statistically consistent under the GTR+MSC model, but in this study we focus on pipelines that operate by first estimating gene trees and then combining these estimated gene trees using a summary method. More specifically, we will restrict the discussion to pipelines that use “coalescent-based” summary methods, as follows:

Definition 3: A **coalescent-based summary method** is a method that estimates the species tree by combining gene trees, and which converges in probability to the true species tree as the number of true gene trees sampled from the distribution defined by the species tree increases.

Examples of coalescent-based summary methods include MP-EST [15], ASTRAL [16, 17], STAR [13] and NJst [18]. Coalescent-based analyses of biological datasets typically use this kind of pipeline, since they can be computationally more efficient than other types of coalescent-based analyses (for example, methods like *BEAST [19] that co-estimate the gene trees and species tree).

Thus, we focus the discussion in this study on phylogenomic pipelines that have the following basic structure:

- Step 1: a gene tree is estimated for each locus
- Step 2: the gene trees are combined into a species tree using a coalescent-based summary method.

While many studies have explored the statistical properties of coalescent-based summary methods given true gene trees, here we focus on their use when the input is a set of sequence alignments for multiple loci. In this context, proofs of statistical consistency for GTR+MSC phylogenomic pipelines have taken the following form [20]. First, gene trees are estimated using a statistically consistent method, such as GTR maximum likelihood, and we assume that the sequences for each locus are long enough that the true gene tree is computed with high probability. Then, the species tree is estimated using a coalescent-based summary method. Thus, the proofs of statistical consistency under the GTR+MSC model for common coalescent-based summary methods (e.g., ASTRAL, MP-EST, NJst, etc.) have explicitly or implicitly assumed that true gene trees are given as input to the summary method.

Little mathematical theory has been proven about the impact of gene tree estimation error on coalescent-based summary methods; for example, it is not known whether standard summary methods will converge to the true species tree given a large enough number of gene trees, if each gene tree is estimated from bounded length sequences [20]. Furthermore, empirical studies suggest that summary methods are impacted by gene tree estimation error, and can produce less accurate estimated species trees than concatenation when gene tree estimation error is high enough (see [20–24] for examples of these studies on summary methods and further discussion). In a genome-scale analysis, it is unlikely that all the loci will have substantial phylogenetic signal, and so this vulnerability to gene tree estimation error means that coalescent-based summary methods may not be highly accurate techniques for estimating species trees from genome-scale data. This is particularly problematic when the sequences for each locus are kept short to diminish the probability of intra-locus recombination (which violates the assumptions of the multi-species model), since short sequences will tend to have insufficient phylogenetic signal to provide full resolution of the gene trees; see [22, 24, 25] for discussion about this important issue.

In [23], we developed a technique we called “Statistical Binning” to improve species tree estimation using phylogenomic pipelines based on coalescent-based summary methods. Statistical binning partitions the genes into sets based on a heuristic to evaluate “combinability”, concatenates the gene sequence alignments within each set into a “supergene alignment”, and then estimates a “supergene tree” on the supergene alignment using a fully partitioned maximum likelihood analysis. The newly estimated supergene trees are then used by the preferred coalescent-based summary method to compute a species tree on the dataset. As shown in [23], statistical binning improved the estimation of gene trees and gene tree distributions, and this resulted in improved estimates of the species tree topology and branch lengths when species trees were computed using MP-EST with multi-locus bootstrapping (MLBS). Furthermore, when used with statistical binning, MP-EST was almost always at least as accurate as concatenation (more accurate than concatenation when the ILS level is high, and only less accurate than concatenation for very low levels of ILS). Finally, MP-EST used with statistical binning was used to compute a species tree on the avian phylogenomic dataset, and this “MP-EST*” tree was nearly identical to the concatenation analysis we obtained; the MP-EST* and concatenation trees were presented in [26] as the two major hypotheses for the avian phylogeny.

Thus, Mirarab *et al.* proposed a new type of phylogenomic species tree estimation pipeline that has four steps instead of two (where the extra two steps are partitioning the genes into bins based on perceived “incompatibility”, and computing supergene trees for each bin using a fully partitioned maximum likelihood analysis). This pipeline, which we referred to as “statistical binning”, showed very promising results when used with MP-EST. However, we did not address the theoretical properties of these pipelines, we only examined model trees with 37 or more species, and we only analyzed one coalescent-based summary method, MP-EST.

In this paper, we report on an extended evaluation of statistical binning. Specifically,

- We provide a proof of statistical inconsistency under GTR+MSC for pipelines based on the original protocol for statistical binning presented in [23].
- We describe a variant of statistical binning that we call “weighted statistical binning”, and provide a proof of statistical consistency under GTR+MSC for pipelines based on weighted statistical binning.
- We evaluate the impact of statistical binning (both weighted statistical binning and the original unweighted statistical binning technique) on biological and simulated datasets under the GTR+MSC model. We examine pipelines using two coalescent-based summary methods, ASTRAL and MP-EST. We include results on simulated and biological datasets studied in [23], and also on additional simulated datasets with 10 and 15 taxa.

The study shows that weighted and unweighted statistical binning have very similar results across most datasets, and also that both ASTRAL and MP-EST tend to improve in accuracy when used with binning. However, there was one condition (characterized by a very high level of ILS, low average bootstrap support for the gene trees, and only ten species) in which statistical binning reduced accuracy for both MP-EST and ASTRAL. Thus, this study shows that binning is often beneficial, but also that there are some conditions under which binning can increase rather than decrease species tree error. Finally, we conclude with suggestions for further research.

### Weighted Statistical Binning

The statistical binning technique presented in [23] operates as follows. The input is a multiple sequence alignment on each of *p* given genes, and a user-specified “threshold support” value *B* < 1. The role of the threshold *B* is to specify which branches in the gene trees are considered reliable, and which ones have support that is so low that the branches may be due to estimation error. Therefore, if the trees on two genes differ only in their low support edges, the differences are considered potentially consistent with estimation error, and the two genes are considered “combinable” or “compatible”.

Statistical binning computes maximum likelihood (ML) gene trees and bootstrap support on the branches for each gene, and then uses a simple heuristic based on bootstrap support values so that two genes can only be in the same bin if their ML gene trees do not have conflicting branches, each with bootstrap support of at least *B*. This is the combinability test, so that two genes are not considered combinable if they have highly supported conflicting branches, and otherwise are considered combinable. (Equivalently, two genes are combinable if their ML gene trees, after collapsing all branches with support less than *B*, share a common refinement.) Finally, because pairwise compatibility ensures setwise compatibility [27], if a set of gene trees *can* be all put in the same bin, then there is a tree that combines all the highly supported branches in any of the trees in the set.

#### Computing and using the incompatibility graph to bin the genes

The first step in statistical binning creates a graph based on the input, and uses a graph-theoretic optimization to bin the genes into subsets. Each gene is represented by a single vertex in the graph, and an edge is placed between two genes if their gene trees are not combinable, based on the heuristic described above. Determining if two genes are combinable can be computed in linear time [28], and so this graph, which we call the incompatibility graph, can be computed in time linear in the number of taxa and quadratic in the number of genes.

Since longer sequences tend to produce more accurate gene trees, having the bins be as large as possible is desirable; this is accomplished indirectly by seeking a coloring with as few colors as possible (i.e., a minimum vertex coloring), which is an NP-hard problem [29]. However, summary methods, such as MP-EST, use the distribution of the gene trees to estimate the species tree. Assuming gene tree reconstruction error only results in low-support branches, binning the genes so that the bins have nearly the same size means that the supergene tree frequency will be close to the true gene tree distribution (assuming that binning combines genes with the same tree, and that we can compute correct supergene trees). Note also that with such a constraint, frequent true gene tree topologies will be represented in several bins, while each of the rarest gene trees will be represented in a smaller number of bins (and perhaps in only one bin). Therefore, the objective is a coloring of the vertices, using a small number of colors, so that every color class contains about the same number of colors. To achieve such a coloring, [23] modified the Brélaz heuristic [30] for minimum vertex coloring, so that during the greedy coloring, a node is added to the smallest bin for which it has no conflicts. This coloring produces a partitioning of the vertices of the graph into subsets based on the color classes; thus, all vertices with the same color are in the same bin.

#### Computing a supergene tree for each bin

Once the vertex coloring is computed, the genes in a given color class form a bin, and their alignments are concatenated into a supergene alignment. Then, a maximum likelihood tree is computed (perhaps with bootstrapping) on each supergene alignment. For estimating supergenes, we use a *fully partitioned analysis* where each gene is assigned a separate partition, and all numeric model parameters are allowed to differ between partitions. We call the trees that are computed on the supergene alignments “supergene trees”. Because using a fully partitioned analysis is key to the theoretical guarantees of statistical binning, we specifically discuss this step in the pipeline.

Concatenated ML analyses of alignments from different loci can be performed in many ways, but their theoretical properties depend on the details of how they are performed, and in particular whether they are performed using an unpartitioned analysis, or a partitioned analysis. In an unpartitioned analysis, all the sites in the concatenated alignment are assumed to evolve down a single model tree (i.e., topology and numeric parameters), and the model tree maximizing the likelihood is sought for the matrix. In contrast, fully partitioned analyses of concatenated alignments assume that the different loci all evolve down the same tree topology, but allow the different parts within the concatenated alignment to have different values for all of the numeric parameters of the model. In the context of the GTR model, a fully partitioned maximum likelihood analysis would allow each locus to have its own 4 × 4 substitution matrix and gene tree branch lengths. Thus, if there are 10 loci within the concatenated alignment, a single tree topology is returned, but also ten different lengths for each branch, and ten different 4 × 4 substitution matrices. Fully partitioned and unpartitioned maximum likelihood analyses can result in different trees, and these analyses have very different theoretical properties; see the example provided in the Methods section, below.

#### Applying summary methods to the supergene trees

**Fig 1:**
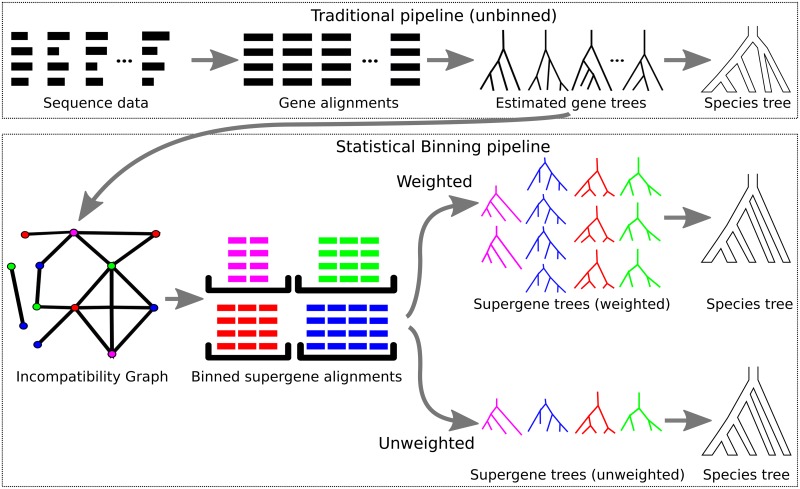
Pipeline for unbinned analyses, unweighted statistical binning, and weighted statistical binning. The input to the pipeline is a set of sequences for different loci across different species. In the traditional pipeline, a multiple sequence alignment and gene tree is computed for each locus, and then these are given to the preferred coalescent-based summary method, and a species tree is returned. In the statistical binning pipeline, the estimated gene trees are used to compute an incompatibility graph, where each vertex represents a gene, and an edge between two genes indicates that the differences between the trees for these genes is considered significant (based on the bootstrap support of the conflicting edges between the trees). The vertices of the graph are then assigned colors, based on a heuristic for balanced minimum vertex coloring, so that no edge connects two vertices of the same color. The vertices with a given color are put into a bin, and the sequence alignments for the genes in a bin are combined into a supergene alignment. A (supergene) tree is then computed for each supergene alignment using a fully partitioned analysis. In the unweighted binning approach (presented in [23]), these supergene trees are then given to the preferred summary method, and a species tree is returned. In the weighted binning approach presented here, each supergene tree is repeated as many times as the number of genes in its bin, and this larger set is then given to the preferred summary method.

The supergene trees are used by a coalescent-based summary method (e.g., MP-EST) to estimate the species tree. In other words, by recalculating the gene trees, statistical binning changes the input to the coalescent-based summary method. Hence, statistical binning is a technique to re-estimate gene trees used within the coalescent-based pipeline for species tree estimation, as shown in Fig 1.

#### Theoretical properties of pipelines based on statistical binning

Theorem 3 shows that the use of statistical binning within a phylogenomic pipeline is not statistically consistent under the GTR+MSC model. The failure to be statistically consistent occurs because we do not *require* that the bins be equally sized; hence, the distribution on supergene trees can be different from the true gene tree distribution.

However, a simple variation of the technique, which was suggested in [23], corrects this problem. We keep the first step of statistical binning the same (i.e., we compute the same incompatibility graph and then use the same heuristic for balanced minimum vertex coloring), and we compute the same set of supergene trees. However, at this point we replicate every supergene tree so that it appears as many times as the number of genes in its bin. For example, if we begin with 100 genes, and obtain 20 bins, then the original statistical binning technique would produce 20 supergene trees that would be given to MP-EST to analyze. In this modified technique, if we begin with 100 genes, we end up with 100 supergene trees (although some supergene trees will be identical). We call this technique “weighted statistical binning”, and refer to the original technique proposed in [23] as “unweighted statistical binning”. We prove that the use of weighted statistical binning is statistically consistent in Theorem 2.

Fig 1 describes the three possible pipelines (unbinned, unweighted binned, and weighted binned) for use with a summary method. In the unbinned analysis, each gene is analyzed independently, a gene tree is estimated for each gene, and then a summary method, such as MP-EST, uses the gene trees to estimate the species tree. In both the weighted and unweighted binned analyses, the gene trees are computed independently, and then the incompatibility graph is formed with one vertex for each gene. In the shown example, there are 12 genes, and so the graph has 12 vertices. The 12 vertices of the incompatibility graph are then assigned colors, with two vertices colored purple, three vertices colored green, three vertices colored red, and four vertices colored blue. Note that no two vertices of the same color have an edge between them. For each color class, the sequence alignments for the associated genes are concatenated into one long supergene alignment, and a supergene tree is computed on the supergene alignment using a fully partitioned maximum likelihood analysis. After this point, the weighted and unweighted binning methods have different strategies. In the unweighted binning method, exactly one copy of each supergene tree is given as input to the summary method, but in the weighted binning method multiple copies of the supergene trees are given as input. Hence, in this example, MP-EST analyzes only four supergene trees in the unweighted binning pipeline, but it analyzes 12 supergene trees in the weighted binning pipeline.

By design, if the bin sizes are exactly the same, then the statistical binning pipelines produced using weighted and unweighted statistical binning produce the same results; hence, these two approaches can only produce different results when the binning is unbalanced.

### Experimental Study

#### Datasets

We use the avian and mammalian simulated datasets studied in [23] (each based on MP-EST analyses of biological datasets, and having at least 37 taxa) and two other collections of simulated datasets with 10 and 15 taxa. The simulated datasets range from moderately low ILS (the lowest ILS mammalian condition) to extremely high ILS conditions (the higher ILS 10-taxon and 15-taxon model conditions), and range in terms of average gene tree bootstrap support (from very low to moderately high). Thus, the simulated datasets provide a range of conditions in which we explore the impact of statistical binning. We also analyzed two biological datasets (a 48-species avian dataset and a 37-species mammalian dataset) studied in [23].

We used biologically-based simulated datasets that were studied in [23], and are based on species trees estimated using MP-EST on the avian dataset of [26] and the mammalian dataset of [31]. In the avian simulation, the markers vary in sequence length (250bp, 500bp, 1000bp, and 1500bp) in order to produce bootstrap support values similar to those we observed in the biological dataset. In the mammalian simulation, we again explored the impact of phylogenetic signal by varying the sequence length (250bp, 500bp, and 1000bp) for the markers. In both cases, three levels of ILS are simulated by multiplying or dividing all internal branch lengths in the model species tree by two, and we also explore various numbers of genes. The mammalian datasets range in ILS level from relatively low (18% average distance between true gene trees and the species tree) for the 2X branch length level to relatively high (54% average distance between true gene trees and the species tree) for the 0.5X branch length level, and the average bootstrap support on the estimated gene trees ranges from low (43%) for the shorter (250bp) sequences to moderately high (79%) for the longest (1000bp) sequences. The avian datasets have higher ILS levels than the mammalian datasets, and range from moderate (35% average distance between true gene trees and the species tree) for the 2X branch length condition to high (59% average distance between true gene trees and the species tree) for the 0.5X branch length condition. The estimated gene trees range in average bootstrap support from very low (27%) for the shortest (250bp) sequences to moderate (60%) for the longest (1500bp) sequences.

We also used a 15-taxon model species tree with a caterpillar-like (also known as a pectinate, or ladder-like) topology, which has 12 short internal branches (0.1 in coalescence units) in succession, a condition that gives rise to high levels of ILS [8, 32]. Ultrametric gene trees were simulated down this tree using the multi-species coalescent process (see Methods). Unlike the biologically-based model conditions, no transformations of branch lengths were used, and therefore, gene trees follow a strict molecular clock. Sequence data were simulated down each gene tree, and we built four model conditions by trimming gene data to 100 or 1000 sites, and by using 100 or 1000 genes. This dataset is very homogeneous since all 10 replicates we simulated are based on the same species tree, and gene trees differ in topology and branch length only due to the coalescence process. The 15-taxon datasets have very high ILS levels (82% average topological distance between true gene trees and the species tree), and so represent a rather extreme condition. The gene trees estimated on the shorter sequences (100bp) had only 35% average bootstrap support, and the combination of very high ILS and very low average bootstrap support represents a very challenging condition. Gene trees estimated on the longer sequences have better average bootstrap support (70%), and so represent a somewhat easier condition.

We also generated 10-taxon simulated datasets using simPhy [33]. In this simulation protocol, we simulated a different species tree for each replicate, and simulated 200 gene trees for each species tree using the multi-species coalescent process. We simulated two model conditions, one with very high ILS and another with somewhat lower (but still high) ILS. The simPhy procedure uses a host of various distributions to make the gene trees heterogeneous in various aspects, such as sequence lengths, deviation of branch lengths from the strict molecular clock, and rate variation across different genes. We used these gene trees to simulate sequence data with 100 sites using Indelible [34]. Therefore, our 10-taxon datasets are very heterogeneous: different replicates have different species trees, and within each replicate, various genes have different rates of evolution. The ILS levels of the 10-taxon datasets range from moderately high (40% average distance from true gene trees to the species tree) for the “lower ILS” condition to extremely high (84% average distance) for the “higher ILS” condition. The average bootstrap support on the estimated gene trees ranged from 37% for the higher ILS condition to 45% for the lower ILS condition, and so both have very poor average bootstrap support. Thus, the 10-taxon and the 15-taxon datasets with short sequences represent the hardest model conditions in that they have very high ILS and very low average bootstrap support.

The simulated datasets we studied varied in many respects (sequence length per locus, whether the sequence evolution is ultrametric or not, and the ILS level). Table 1 presents data about the ILS level, as reflected in the average topological distance between the true gene trees and the true species tree. Note that two of the model conditions (the 10-taxon higher ILS and 15-taxon datasets) have extremely high ILS, reflected in average topological distances between the true gene trees and the species tree. In fact, most of the model conditions have high ILS levels (with 30% or more average topological distance between the true gene trees and the species tree), and only one model condition has low levels of ILS (the mammalian datasets with 2X branch lengths, which have 18% average topological distance between the true gene trees and true species tree). It is likely that the “1X” ILS levels for the mammalian and avian simulated datasets are larger than the ILS levels for the respective biological datasets, since the model trees that were used to generate these data are based on MP-EST analyses of the datasets, and results in [23] suggest that MP-EST estimations tend to under-estimate branch lengths, and hence inflate estimated ILS levels.

**Table 1:** Topological discordance between true gene trees and true species tree. For each collection of simulated datasets (defined by the type of simulation and the ILS level), we show the average topological distance between true gene trees and the species tree.

Dataset	ILS level	Discordance (%)
Avian	2X	35
Avian	1X	47
Avian	0.5X	59
Mammalian	2X	18
Mammalian	1X	32
Mammalian	0.5X	54
10-taxon	Lower ILS	40
10-taxon	Higher ILS	84
15-taxon	High ILS	82

#### Methods

We computed coalescent-based species trees using summary methods with MLBS gene trees in three ways: without binning, with weighted statistical binning and with unweighted statistical binning. Our main focus is on MP-EST, but we explore results with ASTRAL on a subset of the data. ASTRAL estimates species trees given unrooted gene trees, and can analyze very large datasets (such as the plant transcriptome dataset with approximately 100 species and 600 loci [35]); hence, ASTRAL can analyze larger datasets than MP-EST, and so understanding the impact of binning on ASTRAL’s accuracy is of practical importance.

We perform statistical binning using both weighted and unweighted pipelines and using two support thresholds (*B*): 50% and 75%. Due to the extremely large computational effort involved, on our two large biologically-based simulated datasets, we explore one threshold for most of our results; we follow the protocol used in [23] and set *B* = 50% for the avian datasets, and *B* = 75% for the mammalian datasets. However, we also study the impact of *B* on one model condition for avian and mammalian datasets.

We compute gene trees and concatenation species trees using RAxML [36] maximum likelihood. For estimating supergene trees, we use fully partitioned RAxML analyses (using the −*M* option to vary branch lengths across genes) for smaller (10- and 15-taxon) simulated datasets and for all biological analyses. However, since partitioned analyses are expensive, we use unpartitioned analyses to compute supergene trees for our studies on the avian and mammalian simulated datasets (because these studies are very extensive). We compare results using coalescent-based summary methods to concatenation, also using unpartitioned maximum likelihood. Note that the binned methods and the concatenation analysis would potentially become more accurate if fully partitioned analyses were employed.

#### Measurements

For the simulated datasets, we explore species tree accuracy with respect to the true (model) species tree topology (the missing branch rate, or false negative rate (FN)) and branch lengths, and also examine the branch support of both true positive and false positive branches. We also explore the error in the estimated gene trees and gene tree distribution estimated using binning (weighted and unweighted), compared to unbinned analyses. We analyze these simulated datasets using weighted statistical binning with MP-EST and ASTRAL, to determine if there are differences between weighted and unweighted statistical binning. Since ASTRAL does not produce branch lengths, we only use MP-EST to evaluate branch length estimation. In addition, we examine the bootstrap support on the branches of estimated species trees produced using MP-EST, as false positive edges that have low support are not as deleterious as false positive edges with high support. The bootstrap support of estimated species trees was not studied in [23], and so this study provides the first analysis of bootstrap support for MP-EST on these datasets, as well as of the impact of binning on bootstrap support values.

These aspects of phylogenomic estimation are important for different reasons. Species tree topologies indicate which species are more closely related to each other than to others, and so estimating accurate species tree topologies is the most important aspect of phylogenomic estimation. However, the improvement in species tree (coalescent-unit) branch length estimation is also biologically relevant, since these lengths are related to effective population sizes and generation times of ancestral species, and are also used to estimate the amount of ILS in the data. Bootstrap support is important, since low support branches are often ignored, but high support branches are generally assumed to be correct; hence, understanding whether a method returns high support for false positive branches (indicating incorrect relations within a tree) is particularly important. Improvements in estimating the gene tree distribution matter because the accuracy of summary methods depends on an input that captures the correct gene tree distribution.

For the biological datasets, we compare estimated species trees to the literature for each dataset, focusing on whether the estimated species tree violates known subgroups for the phylogeny.

## Results and Discussion

### Biologically-based simulated datasets

#### Gene tree error and gene tree distribution error on avian simulated datasets

We evaluated the impact of statistical binning on gene tree estimation error for the 1X (default ILS) model condition, with sequence lengths varying from 250bp to 1500bp. At the shorter sequence lengths, gene tree estimation error was reduced substantially (from 79% to 57% for 250bp, and from 69% to 57% for 500bp) (S1 Table). Gene tree estimation error was reduced slightly at 1000bp (from 55% to 51%) and even less at 1500bp (from 46% to 45%). Hence, when gene tree estimation error is high due to insufficient sequence length, then binning reduces gene tree estimation error, but binning has little impact on gene tree estimation error when the sequences are long enough.

**Fig 2:**
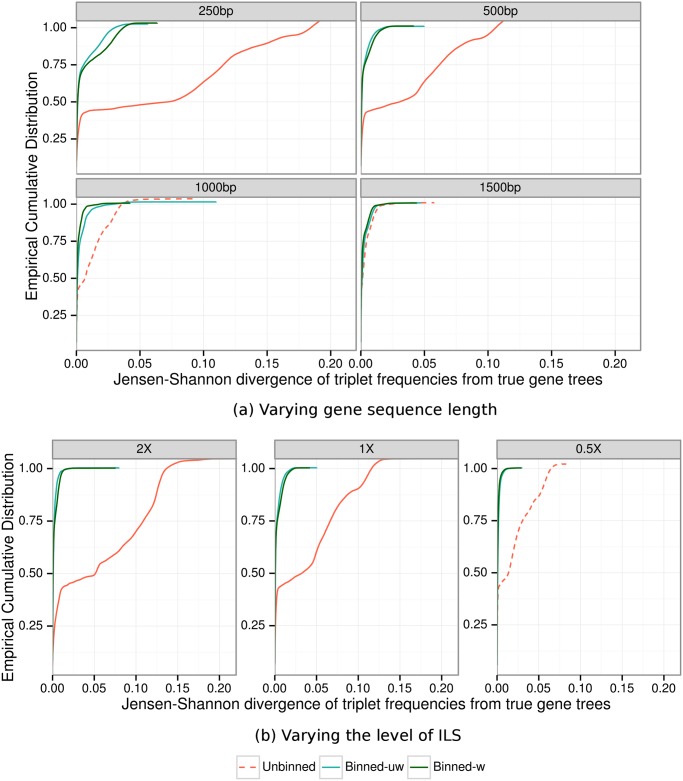
Divergence of estimated gene tree (triplet) distributions from true gene tree distributions for MP-EST analyses of simulated avian datasets. In (a), we vary the gene sequence length (250bp genes have the highest error, and 1500bp has the lowest error) and explore 1000 genes under default ILS levels, and in (b) we vary the amount of ILS and fix the number of genes to 1000 and sequence length to 500bp. True triplet frequencies are estimated based on true gene trees for each of the $\left(\begin{array}{c} n\\ 3 \end{array}\right)$ possible triplets, where *n* is the number of species. Similarly, triplet frequencies are calculated from estimated gene/supergene trees. For each of these $\left(\begin{array}{c} n\\ 3 \end{array}\right)$ triplets, we calculate the Jensen-Shannon divergence of the estimated triplet distribution from the true gene tree triplet distribution. We show the empirical cumulative distribution of these divergence scores. The empirical cumulative distribution shows the percentage of the triplets that are diverged from the true triplet distribution at or below the specified divergence level. Results are shown for 10 replicates. We used 50% bootstrap support threshold for binning, and estimated the supergene trees using RAxML with unpartitioned analyses.

We measure the error in estimated gene tree distributions using the deviation of triplet frequencies from the triplet frequency distribution computed using the true gene trees (see Materials and Methods). We express these results using a cumulative distribution over all possible triplets and all replicates; hence, if a curve for one method lies above the curve for another method, then the first method strictly improves on the second method with respect to estimating the gene tree distribution. In Fig 2(a) we show results for 1000 avian genes under default ILS levels, as we vary the sequence length. In Fig 2(b) we show results with 1000 genes of length 500bp, varying the ILS level. In both cases, both weighted and unweighted binning are nearly identical. Weighted and unweighted binning also show nearly identical gene tree distribution errors under other conditions (see S1 Fig). Binning improves the accuracy of estimated gene tree distributions in general, but not for the longest sequences (1500bp). Also, the improvement over unbinned analyses was highest for the lowest ILS level (2X species tree branch lengths), but was high even for the highest ILS level we explored.

#### Species tree estimation error on avian simulated datasets

**Fig 3:**
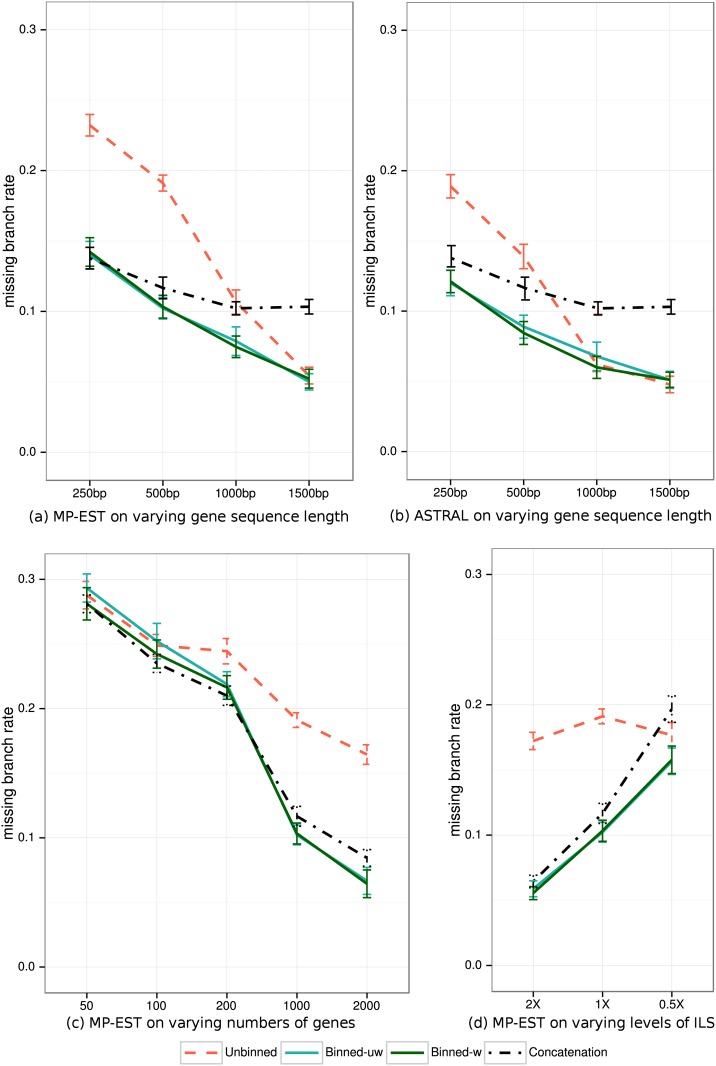
Species tree estimation error (FN) for MP-EST and ASTRAL with MLBS on avian simulated datasets. (a) MP-EST on 1000 genes with varying gene sequence length (controlling gene tree error) and with 1X ILS. (b) ASTRAL on the exact same conditions, (c) MP-EST on varying numbers of genes with fixed default level of ILS (1X level) and 500bp sequence length, and (d) MP-EST on varying levels of ILS and 1000 genes of length 500bp. We show results for 20 replicates everywhere, except for 2000 genes that are based on 10 replicates. Binning was performed using 50% bootstrap support threshold. We estimated the supergene trees, and performed concatenation using RAxML with unpartitioned analyses.

Fig 3 shows results for species tree topology estimation error for analyses of avian genes of different length under the default ILS level using MP-EST and ASTRAL, for varying number of 500bp genes with default ILS using MP-EST, and for 1000 genes of 500bp with varying ILS using MP-EST. Weighted and unweighted statistical binning are essentially identical for both MP-EST and ASTRAL (no statistically significant differences were observed according to a two-way ANOVA test; see Tables 2 and 3), and both reduce species tree estimation error compared to unbinned analyses (differences were always statistically significant with p < 0.001; see Tables 2 and 3).

**Table 2:** Statistical significance test results for choice of binning method on MP-EST. We performed ANOVA to test the significance of the choice of methods (unbinned, weighted binned, unweighted binned, WSB-50: weighted statistical binning using 50% bootstrap support threshold and WSB-75: weighted binning using 75% bootstrap support threshold). For weighted vs. unweighted, we compared 50% bootstrap support threshold for avian, 75% for mammalian, and both 50% and 75% for 15- and 10-taxon datasets. All *p*-values are corrected for multiple hypothesis testing using the FDR correction (*n* = 16). “n.a.” stands for “not available”.

Dataset	Varying parameter	Weighted vs. Unweighted	WSB-50 vs. Unbinned	WSB-75 vs. Unbinned
10-taxon	ILS level	0.96	0.96	0.96
15-taxon	# of genes, seq length	0.96	0.96	**0.04**
Avian	sequence length	0.96	**<0.0001**	n.a.
Avian	ILS level	0.96	**<0.0001**	n.a.
Avian	# of genes	0.91	**<0.0001**	n.a.
Mammalian	# of genes, seq length	0.96	n.a.	**<0.0001**
Mammalian	ILS level	0.96	n.a.	**0.0003**

**Table 3:** Statistical significance test results for choice of binning method on ASTRAL. We performed ANOVA to test the significance of the choice of methods (unbinned, weighted binned, unweighted binned, WSB-50: weighted statistical binning using 50% bootstrap support threshold and WSB-75: weighted binning using 75% bootstrap support threshold). For weighted vs. unweighted, we compared 50% bootstrap support threshold for avian, 75% for mammalian, and both 50% and 75% for 15- and 10-taxon datasets. All *p*-values are corrected for multiple hypothesis testing using the FDR correction (*n* = 14). “n.a.” stands for “not available”.

Dataset	Varying parameter	Weighted vs Unweighted	WSB-50 vs Unbinned	WSB-75 vs Unbinned
10-taxon	ILS level	1	1	0.91
15-taxon	# of genes, seq length	0.91	0.57	**0.008**
Avian	sequence length	0.91	**<0.0001**	n.a.
Avian	sequence length	1	n.a.	0.57
Mammalian	ILS level	0.57	n.a.	**0.0009**
Mammalian	# of genes	0.91	n.a.	**<0.0001**

The largest improvements are for the shortest gene sequences, where error is reduced from 23% to 14% using MP-EST and from 19% to 13% using ASTRAL. The difference between binned and unbinned analyses is lower for 1000bp sequences, and there are no noteworthy differences for 1500bp sequences (sequence length has a statistically significant impact; see Tables 4 and 5). When the number of genes is changed (see Fig 3(c)), the impact of binning on MP-EST ranges from neutral to highly positive, and the largest improvements are for datasets with large numbers of genes (impact of the number of genes is statistically significant; see Table 4). The impact of binning is also significantly impacted by ILS levels (see Table 4), with the largest improvements obtained for lower levels of ILS. In general, binning helps both ASTRAL and MP-EST, but MP-EST tends to be helped more than ASTRAL. For example, with 500bp genes, the error for MP-EST is reduced from 19% to 10% using binning, but error of ASTRAL is reduced from 15% to 9%.

**Table 4:** Statistical significance test results for interaction effects (binning and simulation parameter) on MP-EST. We performed ANOVA to test the significance of whether there is an interaction between the choice of the method (unbinned, weighted binned, unweighted binned, WSB-50: weighted statistical binning using 50% bootstrap support threshold and WSB-75: weighted statistical binning using 75% bootstrap support threshold) and the variable changed in each dataset. For weighted vs. unweighted, we compared 50% bootstrap support threshold for avian, 75% for mammalian, and both 50% and 75% for 15- and 10-taxon datasets. All *p*-values are corrected for multiple hypothesis testing using the FDR correction (*n* = 21). “n.a.” stands for “not available”.

Dataset	Interaction variable	Weighted vs Unweighted	WSB-50 vs Unbinned	WSB-75 vs Unbinned
10-taxon	ILS level	0.99	0.99	0.49
15-taxon	# of genes, seq length	0.99 & 0.99	0.59 & 0.99	0.24 & 0.17
Avian	sequence length	0.99	**<0.0001**	n.a.
Avian	ILS level	0.99	**<0.0001**	n.a.
Avian	# of genes	0.99	**<0.0001**	n.a.
Mammalian	# of genes, seq length	0.99 & 0.99	n.a.	0.99 & 0.38
Mammalian	ILS level	0.15	n.a.	0.15

**Table 5:** Statistical significance test results for interaction effects (binning and simulation parameter) on ASTRAL. We performed ANOVA to test the significance of whether there is an interaction between the choice of the method (unbinned, weighted binned, unweighted binned, WSB-50: weighted statistical binning using 50% bootstrap support threshold and WSB-75: weighted statistical binning using 75% bootstrap support threshold) and the variable changed in each dataset. For weighted vs. unweighted, we compared 50% bootstrap support threshold for avian, 75% for mammalian, and both 50% and 75% for 15- and 10-taxon datasets. All *p*-values are corrected for multiple hypothesis testing using the FDR correction (*n* = 17). “n.a.” stands for “not available”.

Dataset	Interaction variable	Weighted vs Unweighted	WSB-50 vs Unbinned	WSB-75 vs Unbinned
10-taxon	ILS level	0.99	1	0.99
15-taxon	# of genes, seq length	0.99 & 0.99	0.99 & 0.99	0.29 & **0.02**
Avian	sequence length	0.99	**<0.0001**	n.a.
Mammalian	sequence length	0.99	n.a.	0.29
Mammalian	ILS level	0.99	n.a.	0.29
Mammalian	# of genes	0.99	n.a.	0.99

**Fig 4:**
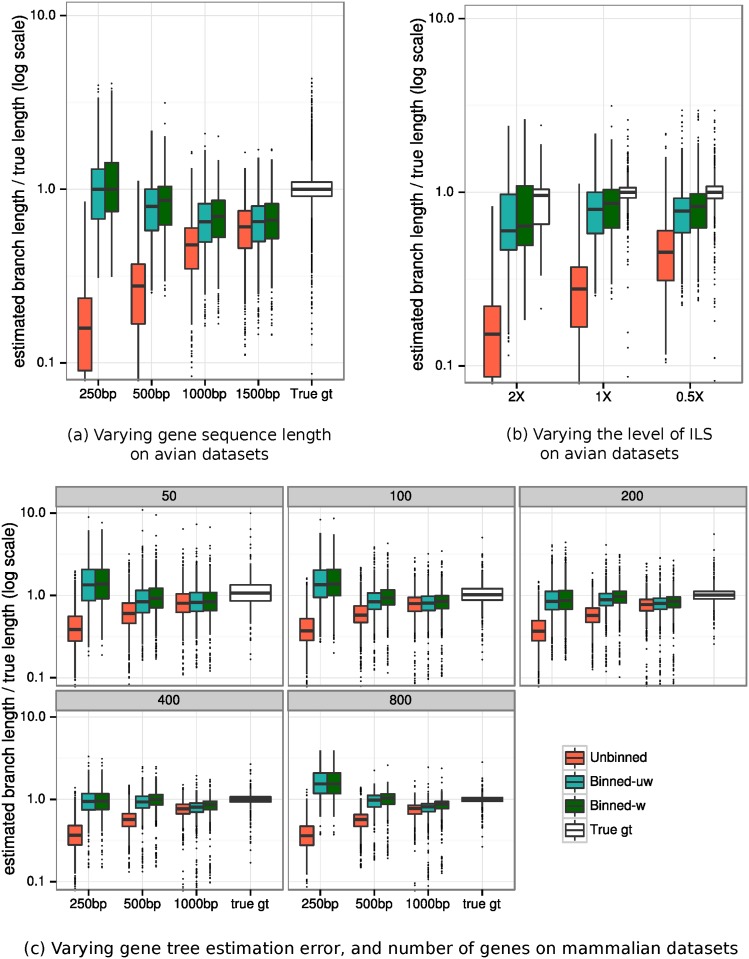
Effect of binning on the branch lengths (in coalescent units) estimated by MP-EST using MLBS on the avian and mammalian simulated datasets. We show the species tree branch length error (the ratio of estimated branch length to true branch length for branches of the true tree that appear in the estimated tree; 1 indicates correct estimation). Results are shown for (a) 1000 avian genes of 1X ILS level with varying gene sequence length, (b) 1000 avian genes of 500bp and with varying levels of ILS, and (c) varying number of mammalian genes and varying sequence length (250bp, 500bp, and 1000bp) with 1X ILS level. Results are shown for 20 replicates. We used 50% and 75% bootstrap support threshold for binning on avian and mammalian datasets, respectively, and estimated the supergene trees using RAxML with unpartitioned analyses.

Fig 4 shows the impact of binning on species tree branch length estimation error on the biologically-based simulations using MP-EST; Fig 4(a) shows results on 1000 genes under default (1X) ILS levels and varying gene sequence length, and Fig 4(b) shows results on 1000 genes of 500bp with varying ILS levels. Branch length estimation accuracy is reported using the ratio of the estimated branch length to the true branch length, for those true branches recovered by the method. Thus, values equal to 1 indicate perfect accuracy, values below 1 indicate under-estimation of branch lengths (and hence over-estimation of ILS), and values above 1 indicate over-estimation of branch lengths (and hence under-estimation of ILS).

Both types of binning (weighted and unweighted) produce nearly identical results with respect to species tree branch length estimation (with a slight advantage for weighted analyses). Unbinned analyses substantially under-estimate branch lengths, but as the sequence length increases, the branch length estimations produced by unbinned analyses improve, so that they are more accurate with 1500bp markers. The most accurate species tree branch length estimation is obtained using true gene trees. Using binning (either type) improves branch length estimation from estimated gene trees, and the improvement is very large for the shorter sequences (Fig 4(a)). When levels of ILS are changed, weighted and unweighted binning are again close (with a slight advantage for weighted), and show little change in branch length estimation with changes in ILS levels; however, unbinned analyses substantially under-estimate branch lengths for the lowest ILS model condition, and then become more accurate (although still under-estimate) with increases in the ILS level. Hence, the biggest improvement obtained by binning is for the lowest ILS (2X branch lengths), and there is less improvement for the highest ILS level (0.5X). The likely explanation for this trend is that MP-EST interprets all discord as due to ILS, and produces a model tree (with branch lengths) that it considers most likely to generate the observed discordance. Hence, MP-EST tree branch lengths will be closer to the correct lengths when the ILS level is very high.

#### Bootstrap support on avian simulated datasets

We explore bootstrap support of trees estimated on simulated avian datasets, as follows. We assign relative quality to each edge in an estimated tree, taking bootstrap support into account. The highest quality edges are the true positive branches with the highest bootstrap support, and the lowest quality edges are the false positive branches with the highest bootstrap support, and all other edges fall in between. We order all the edges by their quality, so that the true positive branches come first (with the high support branches before low support branches), followed by the false positive branches (with the low support branches before the high support branches). Given this ordering, we create figures in which the x-axis indicates the edge quality (from very high to very low, as you move from left to right), and the y-axis indicates the fraction of the edges having at least the quality indicated by the x-axis. Thus, the higher the curve, the better the overall quality of the species tree.

**Fig 5:**
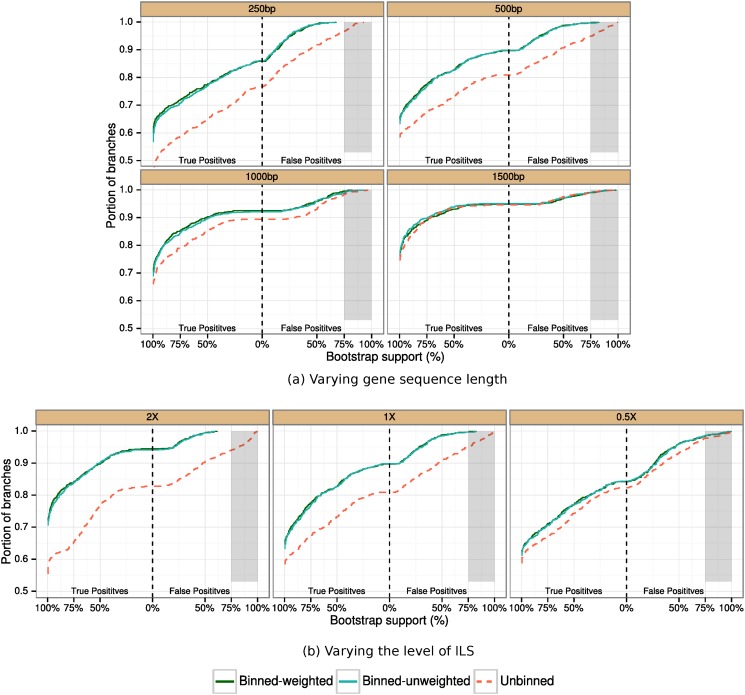
Cumulative distribution of the bootstrap support values (obtained using MLBS) of true positive (TP) and false positive (FP) edges estimated by binned and unbinned MP-EST on avian datasets. In (a) we fix the number of genes to 1000, use default ILS levels, and vary sequence length to control gene tree estimation error, and in (b) we study 1000 genes with 500bp sequence length, and vary ILS levels. To produce the graph, we order the branches in the estimated species tree by their quality, so that the true positives with high support come first, followed by lower support true positives, then by false positives with low support, and finally by false positives with high support. The false positive branches with support above 75% are the most troublesome, and the highly supported false positives are indicated by the grey area. When the curve for a method lies above the curve for another method, then the first method has better bootstrap support. We used 50% bootstrap support threshold for binning, and estimated the supergene trees using RAxML with unpartitioned analyses.

Fig 5 shows results on 1000 avian genes under default ILS and with varying sequence length, and also with 1000 genes of 500bp with varying ILS levels. Both types of binning are nearly identical in terms of their impact on bootstrap support, and both improve bootstrap support; in particular, using binning increases the number of highly supported true positive branches and decreases the number of highly supported false positive branches. However, the sequence length modulates the impact of binning on bootstrap support, so that the largest impact is for the shortest sequences (250bp) and there is no discernible impact for the longest sequences (1500bp). ILS levels also impact how binning affects the bootstrap support, so that the biggest improvement in bootstrap support is obtained for the lowest ILS level (2X branch lengths). The number of genes also impacts the bootstrap support (supporting information S2 Fig). so that the biggest improvement in bootstrap support is obtained for the largest number of genes (2000) (and there is little to no difference between binned and unbinned analyses on 50 or 100 genes); furthermore, weighted and unweighted binning produce very similar bootstrap support values.

#### Comparisons to concatenation on avian simulated datasets

On the shortest 250bp sequences, concatenation matches the accuracy of weighted and unweighted binned MP-EST methods (Fig 3(a)) and is slightly less accurate than both binned ASTRAL trees (Fig 3(b)). As sequence length increases, both types of binning using either ASTRAL or MP-EST become more accurate than concatenation. Unbinned analyses are less accurate than concatenation for shorter sequences and more accurate for longer sequences (the transition point depends on whether ASTRAL or MP-EST is used). Both binned analyses are more accurate than concatenation and unbinned analyses at all ILS levels (Fig 3(d)). Thus, compared to concatenation, binned analyses have their largest advantage on longer gene sequences, higher ILS levels, and higher number of genes.

#### Results on mammalian datasets

**Fig 6:**
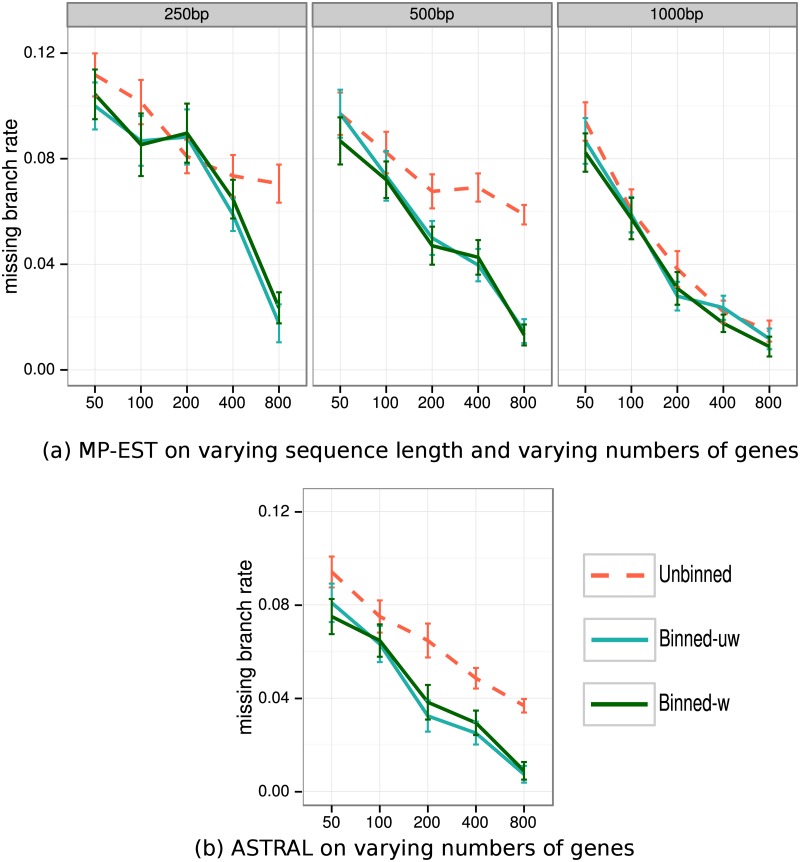
Species tree estimation error for MP-EST and ASTRAL using MLBS on mammalian simulated datasets. We show average FN rate over 20 replicates. (a) Results for MP-EST. We varied the number of genes (50, 100, 200, 400 and 800) and sequence length (250bp (43% BS), 500bp (63% BS) and 1000bp (79% BS)) with default amount of ILS (1X level). (b) ASTRAL on varying numbers of genes with fixed 1X ILS level and 500bp sequence length. We used 50% and 75% bootstrap support threshold for binning on avian and mammalian datasets, respectively, and estimated the supergene trees using RAxML with unpartitioned analyses.

Results on simulated mammalian datasets are similar to analyses of avian datasets. In nearly every condition, both weighted and unweighted binning show very similar results (see Fig 6) and have no statistically significant differences using either ASTRAL or MP-EST (see Tables 2 and 3). As before, we evaluated the impact of statistical binning on gene tree estimation error under the 1X (default ILS) model condition with varying sequence lengths (S1 Table), and observed that binning substantially reduces gene tree estimation error for short sequences (250bp and 500bp) but had little impact on longer sequences (1000bp). Binning improves gene tree distributions, generally with very large improvements, and the improvements decrease with the sequence length and ILS level (S3 Fig). Binning also improves species tree topology estimation (Fig 6 and Tables 2 and 3). The impact appears to depend on the sequence length (binning seems more beneficial for shorter sequences and neutral for longer sequences) and number of genes (binning can dramatically improve species tree topologies given a large number of genes, but can be neutral or even detrimental for a small number of genes), and the choice of summary method (binning helps both ASTRAL and MP-EST, but helps MP-EST more). ILS level also seems to impact relative accuracy (Tables 4 and 5), so that binning seems most helpful for low ILS levels, and less helpful for high ILS levels (S4 Fig). However, the effects of number of genes, sequence length, and the ILS level were not statistically significant for this dataset (Tables 4 and 5).

As observed in the avian simulations, unbinned analyses substantially under-estimate species tree branch lengths (Fig 4(c) and S5 Fig). Both weighted and unweighted binning produce nearly identical branch lengths for all sequence lengths, number of genes, and ILS levels, and both types of binning come closer to the true branch lengths than unbinned analyses. Finally, both weighted and unweighted binning produce nearly identical species tree branch support values, where both match or improve unbinned analyses for all tested numbers of genes, sequence lengths, and ILS levels (S6 and S7 Figs). However, improvements increase with the number of genes and decrease with the sequence length and ILS level.

#### Impact of support threshold *B* on avian and mammalian simulated datasets

**Fig 7:**
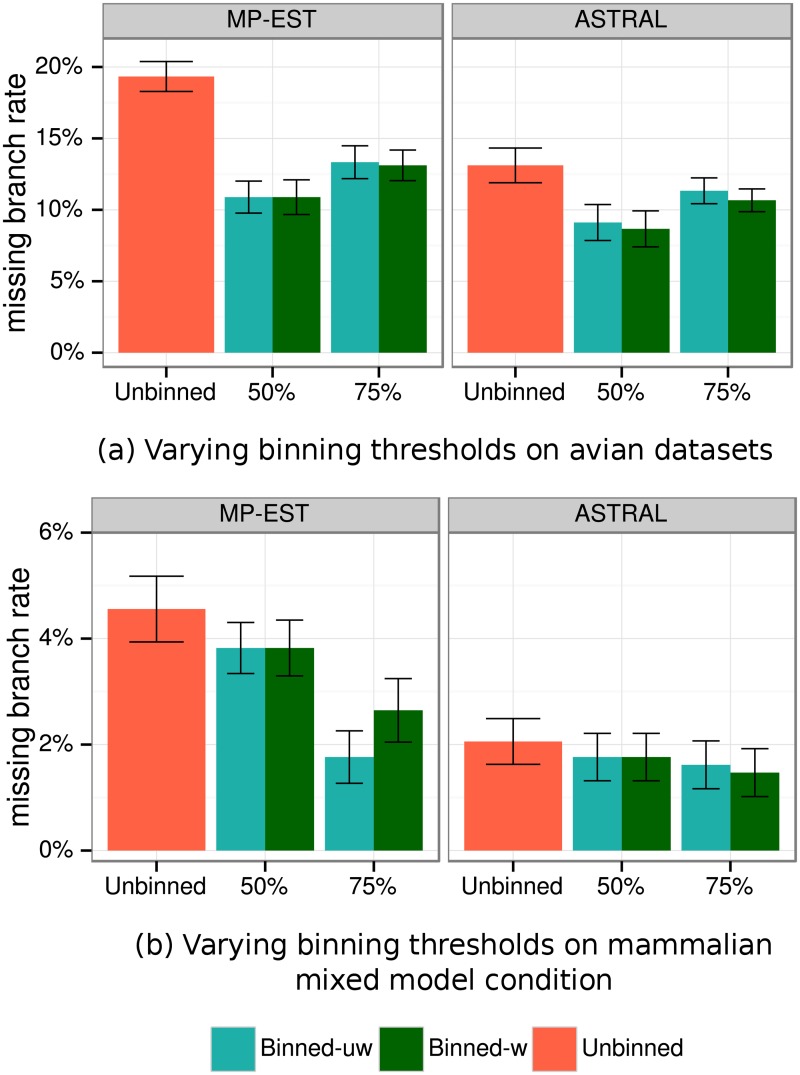
Species tree estimation error for MP-EST and ASTRAL using MLBS on avian and mammalian simulated datasets with two support thresholds (*B*). We show average FN rate for unbinned, and wighted and unweighted binned analyses with both *B* = 50% and *B* = 75%. Results are shown for (a) the avian dataset with 10 replicates of 1000 genes of length 500bp and 1X ILS level, and (b) the mammalian dataset with 20 replicates of 400 mixed genes (200 genes with 500bp and 200 genes with 1000bp) with 1X ILS level.

In addition to varying model conditions, we use a single avian and a single mammalian model condition to study the impact of the support threshold *B* on binning (Fig 7). We use a mixed model condition with 200 genes of 500bp and 200 genes of 1000bp for the mammalian dataset, and a model condition with 1000 genes of 500bp for the avian dataset (both with default 1X ILS level).

On the avian dataset, binning is always beneficial, but the impact is larger with *B* = 50% compared to *B* = 75% (Fig 7(a)). For example, unbinned MP-EST has 19% error, and using *B* = 50% reduces the error to 11%, and using *B* = 75% reduces the error to 13%.

On the mammalian mixed data, binning is beneficial in all cases (see Fig 7(b)); however, the extent of the impact depends substantially on both the threshold and the summary method. ASTRAL has high accuracy even without binning, and binning with either threshold has only a small impact on its accuracy. When MP-EST is used, binning with *B* = 50% leads to relatively small improvements in accuracy, whereas *B* = 75% results in much larger improvements. Thus, the choice of the threshold can have an impact, but for the two model conditions we studied here both choices of the threshold are beneficial.

#### Effects of binning on gene tree and species tree error for 15-taxon datasets

We explored the impact of statistical binning on gene tree estimation error using two sequence lengths and two values for *B*, the bootstrap support threshold parameter (S1 Table). For the shorter sequence lengths (100bp), binning increases gene tree estimation error (from 77% to 80% when *B* = 50%, and from 77% to 86% when *B* = 75%). For the longer sequence lengths (1000bp), binning with *B* = 50% has no impact on gene tree estimation error, but using *B* = 75% increases error from 36% to 40%. Thus, statistical binning increases gene tree estimation error for these very high ILS 15-taxon datasets, but the amount of the increase depended on the parameter *B* (with larger increases for *B* = 75% and small increases for *B* = 50%) and sequence length (where the impact on gene tree estimation error is much reduced for the 1000bp alignments).

**Fig 8:**
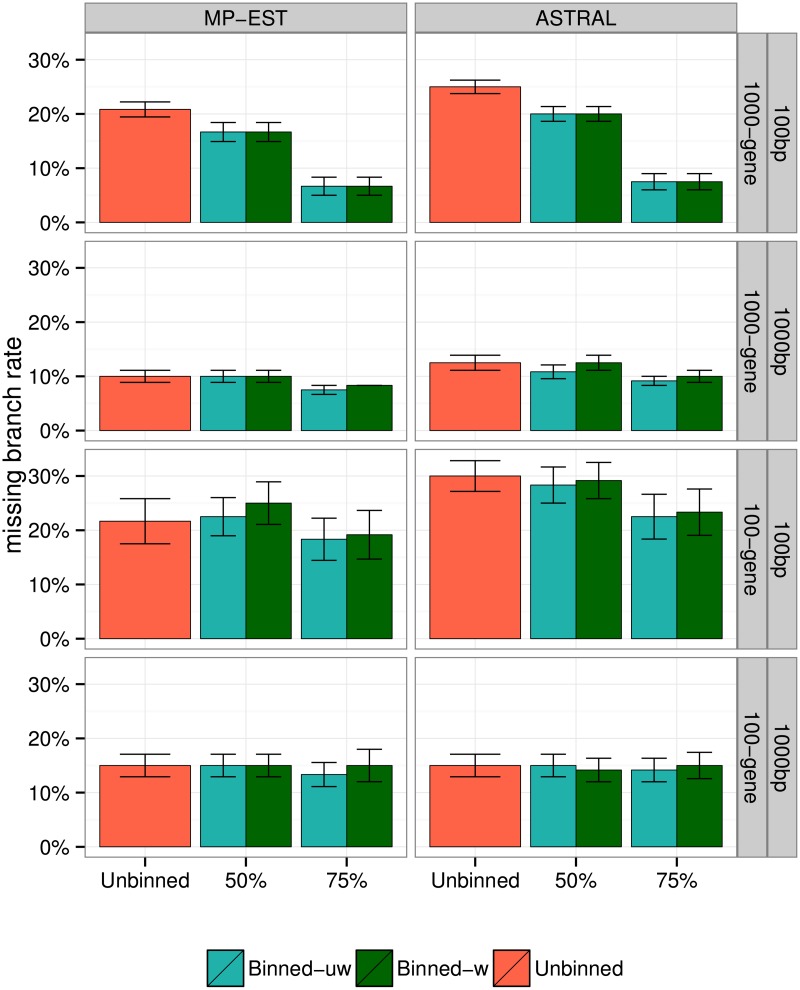
Species tree estimation error for MP-EST and ASTRAL with MLBS on 15-taxon simulated datasets. We show average FN rate over 10 replicates. We varied the number of genes (100 and 1000) and sequence length (100bp and 1000bp). We used 50% and 75% bootstrap support thresholds for binning, and estimated the supergene trees using RAxML with fully partitioned analyses.

Fig 8 shows the impact of weighted and unweighted statistical binning on species tree accuracy for the 15-taxon dataset. We apply statistical binning with two support thresholds (50% and 75%), and we use both MP-EST and ASTRAL as the summary method. In all cases, weighted and unweighted binning have similar accuracy, with no statistically significant differences (Tables 2 and 3). The relative accuracy of unbinned and binned analyses depends on the support threshold, so that with *B* = 50%, there are no statistically significant differences, but with *B* = 75%, binning significantly improves accuracy (*p* = 0.04 for MP-EST and *p* = 0.008 for ASTRAL; Tables 2 and 3). The extent of the improvements seems larger for more genes and smaller alignments, but the impact of these factors are not statistically significant for MP-EST (*p* = 0.24 and *p* = 0.17 respectively) and only impact of sequence length was significant for ASTRAL (*p* = 0.02; Tables 4 and 5). The biggest gains are obtained when the 75% threshold is used with 1000 genes of 100bp, where binning reduces the error of MP-EST from 21% to only 7%. Thus, the choice of the threshold can matter, and on this dataset, the effects of binning can range from neutral to highly beneficial, depending on the threshold used, number of genes, and gene sequence length.

#### Effects of binning on species tree error for 10-taxon datasets

**Fig 9:**
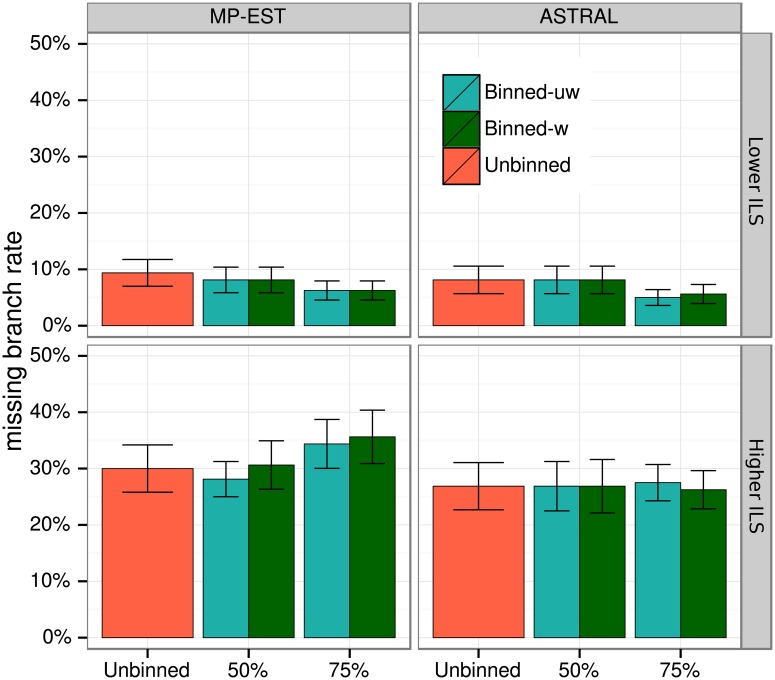
Species tree estimation error for MP-EST and ASTRAL with MLBS on 10-taxon simulated datasets. We show average FN rate over 20 replicates. We varied the amount of ILS and fixed the number of genes to 200 and gene sequence length to 100bp. We used 50% and 75% bootstrap support thresholds for binning, and estimated the supergene trees using RAxML with fully partitioned analyses.

Fig 9 shows the impact of binning on species tree accuracy on the 10-taxon datasets with two choices of the threshold *B* for the statistical binning pipeline (*B* = 50% and 75%), two choices of the summary method (MP-EST and ASTRAL), and two levels of ILS (high and very high). No statistically significant differences are observed on these data between weighted and unweighted binning, or between weighted binning and unbinned analyses (see Tables 2 and 3); nevertheless, some patterns can be observed in terms of the average error (Fig 9). Both weighted and unweighted statistical binning are close to neutral (regardless of the choice of method or level of ILS) when applied with a 50% threshold. When the 75% threshold is used, the impact of binning depends on the level of ILS: binning improves accuracy with low ILS levels and reduces accuracy with high ILS levels, especially when MP-EST is used, but these differences are not statistically significant (Tables 2 and 3).

### Analysis of biological datasets

We compared weighted and unweighted binning of MP-EST and ASTRAL on MLBS gene trees on the avian and mammalian biological datasets studied in [23].

**Fig 10:**
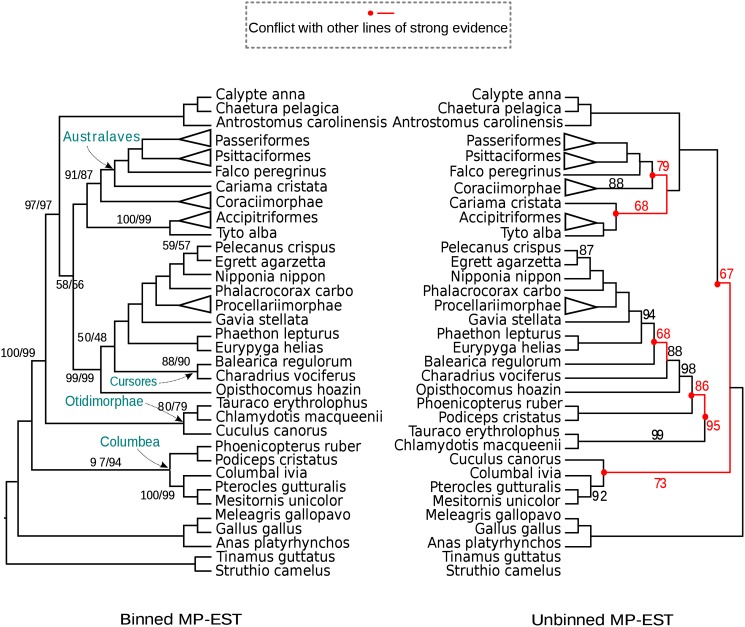
Trees computed on the avian biological dataset using MP-EST on MLBS gene trees. We show results with weighted and unweighted binning (left), and unbinned analyses (right). We used 50% bootstrap support threshold for binning. Supergene trees were estimated using fully partitioned analyses. MP-EST with weighted and unweighted binning returned the same tree. The branches on the binned MP-EST tree are labeled with two support values side by side: the first is for unweighted binning and the second is for weighted binning; branches without designation have 100% support. Branches in red indicate contradictions to known subgroups.

Results for MP-EST on these datasets showed the following trends. First, for the avian dataset, there are no topological differences between MP-EST trees estimated using weighted or unweighted statistical binning, and extremely small differences in branch support (less than 3%; see Fig 10). Thus, although [26] only explored unweighted statistical binning with MP-EST, the main conclusions they drew about the evolutionary history of modern birds are also found in the weighted statistical binning analysis using MP-EST. The unbinned MP-EST analysis violates several subgroups established in the avian phylogenomics project and other studies (indicated in red in Fig 10), but the binned MP-EST analyses do not violate any of these subgroups. Of these violated subgroups, the failure of the unbinned MP-EST analysis to recover Australaves is the most significant, since it has been recovered in many prior analyses [37–40]. On the mammalian dataset, weighted and unweighted MP-EST again produce the same exact tree, with small differences in support (less than 3%; see S11 Fig). The unbinned MP-EST tree, however, has one topological difference (the position of treeshrews; compare S10 and S11 Figs). with binned analyses, as discussed in [23].

Results for ASTRAL on the biological datasets show generally similar trends. Unbinned ASTRAL on the avian dataset (S8 Fig) recovers Australaves and hence is more in line with the prior literature than unbinned MP-EST; however, just like unbinned MP-EST, the unbinned ASTRAL does not recover some key clades recovered by concatenation and other analyses reported in [26]. Using weighted and unweighted statistical binning with ASTRAL on the avian dataset produces almost identical results, and are also almost identical to the binned MP-EST tree (the only change is the position of hoatzin which has low support in all trees; see supporting information S9 Fig). On the mammalian dataset, trees produced by binned ASTRAL analyses with weighted or unweighted binning pipelines are topologically identical to each other, and to the tree produced by the unbinned analysis, and have rather small differences in bootstrap support (see S12 Fig). Binned and unbinned ASTRAL analyses and binned MP-EST analyses all put treeshrews as sister to Glires, while unbinned MP-EST puts them as sister to primates. The placement of treeshrews is of substantial debate, and so the differential placement is of considerable interest in mammalian systematics.

Overall, results on the two biological datasets show that weighted and unweighted statistical binning analyses produced identical species trees and nearly identical branch support values; furthermore, these binned analyses were more congruent with established subgroups than unbinned analyses.

### Summary of observations

Across all our analyses, results for both ASTRAL and MP-EST are very similar with respect to how they responded to statistical binning. Weighted statistical binning produces nearly identical results to unweighted statistical binning on the biologically-based simulated datasets, and topologically identical results (with very similar bootstrap support values) on the biological datasets we explored in this study, and so this study generally supports the conclusions about statistical binning in [23]. In addition, because weighted and unweighted statistical binning produce topologically identical trees on the avian dataset, this study supports the findings about the avian phylogeny reported in [26]. The fact that weighted and unweighted binning typically produced similar results is not surprising, since the unweighted binning technique strives to create “balanced” bins as much as possible, and largely achieves this on the datasets we explored. Furthermore, if the bins produced by statistical binning have *exactly* the same size, then pipelines based on weighted and unweighted statistical binning will produce the same species tree, since the distributions of gene trees they produce will be identical. Since the bin sizes produced using our heuristic for balanced minimum vertex coloring are close to balanced, this explains why we observed very small differences between weighted and unweighted statistical binning in these analyses.

Under most of the model conditions we studied, both weighted and unweighted statistical binning improved the estimation of gene tree topologies, gene tree distributions, species tree topologies and branch lengths, and bootstrap support (so that statistical binning increases bootstrap support for true positive edges, and reduces the number of highly supported false positives), compared to unbinned analyses. These improvements are largest when gene sequence alignments have low phylogenetic signal, the gene trees exhibit at most moderately large ILS levels, or there are many genes.

The impact of statistical binning on the 15-taxon datasets is somewhat different than for the biologically-based simulations. Gene tree estimation accuracy is reduced for both sequence lengths (though the impact is small for the longer sequence lengths and only substantial for the short sequence lengths with *B* = 75%). Nevertheless, the impact on species tree estimation on these data tends to be neutral, but there are also conditions where binning was beneficial.

On the lower ILS 10-taxon datasets, statistical binning reduces gene tree estimation error, and both weighted and unweighted binning reduce species tree estimation error for *B* = 75%. However, species tree estimation error is unchanged when *B* = 50%.

The results on the higher ILS 10-taxon datasets stand out from the other analyses: statistical binning slightly increases gene tree estimation error when B = 50% but substantially increases gene tree estimation error when B = 75%. Furthermore, while species tree estimation error is not increased for *B* = 50%, when *B* = 75%, the error increases.

The difference in impact for statistical binning in this case is interesting, and points out the significance of how *B* is set. To understand this, note that when *B* is very small, then bin sizes will tend to be very small, since any pair of incompatible branches with support above *B* will be considered to be evidence of statistically significant discord; thus, small settings for *B* produce results that are similar to unbinned analyses. Conversely, very large settings for *B* are more likely to bin genes together, since only the strongest supported conflicting branches will prevent genes from being binned. Therefore, if all the gene trees have low support then statistical binning could tend to produce results that are similar to concatenation. Thus, the choice of the threshold matters.

To better understand the difference in impact of statistical binning on these simulated datasets, it is helpful to consider the ILS levels and gene tree bootstrap support values for these data. As shown in Table 1, the average distance between the true gene trees and the species trees ranges for these datasets from as low as 18% (for the Mammalian 2X collection) to above 80% (for the 10-taxon higher ILS collection and the 15-taxon collection). Fig 3 shows how the effect of statistical binning used with MP-EST is impacted by ILS level on the avian datasets: statistical binning provided an improvement at all ILS levels, with the largest improvement for the lowest ILS level (2X branch lengths) and the smallest improvement on the highest ILS level (0.5X branch lengths). S3–S5 Figs evaluate this issue on the mammalian datasets, and shows large improvements provided by statistical binning under the lowest (2X branch lengths) ILS level, smaller improvements under the middle (1X branch length) ILS level, and then no improvement under the highest (0.5X branch lengths) ILS level. Thus, statistical binning provided an improvement except for a small number of model conditions: some of the 15-taxon conditions (which have discordance of 82%), the higher ILS 10-taxon conditions (which have discordance of 84%), and the highest ILS mammalian condition (which have discordance of 54%). S2 Table shows that the average bootstrap support for the higher ILS 10-taxon datasets is quite low—only 37%. Thus, statistical binning seems to be beneficial when both ILS level and gene tree bootstrap support are not too high, will be neutral when bootstrap support values are high (so little or no binning occurs), but can be detrimental when ILS levels are extremely high but gene tree bootstrap support is low enough that binning occurs. Thus, one consequence of this study is the suggestion that when ILS levels are very high and the average gene tree bootstrap support is low, then either statistical binning should not be used, or it should be used in a very conservative fashion—with the parameter *B* set very low.

## Conclusions

Because species trees and gene trees can differ, the estimation of species trees requires multiple loci. One approach to estimating species trees from multiple conflicting loci seeks to restrict the set of loci using principled arguments [41], but other approaches that explicitly model the discordance have also been developed. When gene tree discord is due to incomplete lineage sorting, then summary methods, such as MP-EST or ASTRAL, can be used to estimate the species tree by combining gene trees. However, this study, as well as others [16, 21–24, 42], demonstrates that gene tree estimation error impacts species tree estimation, so that species trees estimated using summary methods on poorly estimated gene trees can have low accuracy. The (unweighted) statistical binning technique proposed in [23] improved the accuracy of estimated gene trees, and was shown to improve the accuracy of MP-EST when applied to MLBS gene trees. However, as we proved here, using unweighted statistical binning within a phylogenomic pipeline can be statistically inconsistent under the GTR+MSC model. This is a significant issue.

This study described a simple modification to statistical binning, obtained by replicating each supergene tree by the number of genes in its bin (equivalently, replacing each gene tree from the input set by its recalculated tree, which is the supergene tree for the bin). This modification, which we call “weighted statistical binning” (WSB), is statistically consistent under GTR+MSC model (i.e., as the number of genes and the number of sites for each gene increases, the estimated species tree topology converges to the true species tree topology), and so addresses this drawback. However, the current mathematical theory does not suggest any advantage will be gained using WSB within a phylogenomic pipeline, compared to an unbinned analysis (i.e., the use of the summary method without binning) because when gene sequence length is unbounded, unbinned analyses using summary methods are also statistically consistent. Indeed, the current mathematical theory about standard coalescent-based summary methods does not establish any guarantees in the presence of gene tree estimation error (which is inevitable given limited length), and the same limitation applies to the theory established for WSB. Hence, from a theoretical standpoint, there is no benefit obtained in using WSB—at least not according to the current mathematical theory.

The reason to use WSB within a phylogenomic pipeline is empirical—how it impacts the accuracy of the estimated species trees—and so our study focused on whether WSB tends to increase or decrease the accuracy of summary methods, and how the model conditions impact the relative performance of binned and unbinned analyses.

On the biologically-based simulated datasets, weighted and unweighted statistical binning generally improved estimated gene tree distributions and led to improvements for MP-EST and ASTRAL estimations of species tree topologies. The use of statistical binning with MP-EST also improved estimated species tree branch lengths, increased bootstrap support for true positive edges, and reduced the number of highly supported false positives, compared to unbinned MP-EST analyses. These improvements increased when gene sequence alignments had low phylogenetic signal, the species tree had low ILS, or there were many genes.

The estimation of species tree branch lengths is biologically significant since these lengths are used to infer the amount of ILS in the data. Unbinned MP-EST analyses tended to substantially underestimate branch lengths (and thus over-estimate ILS), but both weighted and unweighted binning reduce this problem and produce branch lengths that are much closer to their true lengths. Since MP-EST tends to over-estimate ILS in the presence of gene tree estimation error, this means that predictions of ILS levels for biological datasets may have been over-estimated. Another consequence of this observation is that the biologically-based model species trees used here and in [23, 43] may have inflated levels of ILS, since they used MP-EST to construct the model species tree. If so, then performance under the lower ILS levels (species tree branch lengths of 2X or larger) might be closer to the biological dataset conditions than the default 1X condition and higher ILS conditions.

The improvement in branch support is biologically relevant, especially since unbinned MP-EST analyses sometimes produced highly supported false positive branches in the presence of poorly estimated gene trees and low levels of ILS, but binning reduced the incidence of these false positive branches with high support.

The results on small numbers of species, and in particular on the higher ILS 10-taxon datasets, show somewhat different trends. While results on the 15-taxon datasets showed binning generally being helpful or neutral, statistical binning ranged from neutral to detrimental on the higher ILS 10-taxon datasets (however, the differences were not statistically significant). Both the higher ILS 10-taxon and 15-taxon datasets had extremely high levels of ILS (the two highest we examined—average topological distance between true gene trees and the true species trees of 84% and 82%, respectively). Given that statistical binning ranged from neutral to highly beneficial for all the other model conditions, these data suggest that statistical binning may not be suitable to datasets with extremely high ILS levels. Clearly, further research is therefore needed to understand the conditions under which binning will be beneficial and where binning may reduce accuracy.

This study also did not examine model conditions in which gene tree estimation error is due to model misspecification, nor other biological causes for gene tree discord, such as gene duplication and loss or horizontal gene transfer. Furthermore, while we examined sequence datasets with varying numbers of sites for each locus (including some with 100bp), even shorter sequences may be needed to avoid loci that include any recombination [24].

This study mainly examined the impact of statistical binning on MP-EST, and examined its impact on ASTRAL only for a subset of the data and only with respect to species tree topology estimation (instead of the full set of criteria). Thus, an important direction for future study is to consider other coalescent-based methods for estimating the species tree from multiple loci. As a simple example, Mirarab *et al.* [43] showed that the accuracy of MP-EST species trees depended on whether MLBS or best maximum likelihood (BestML) gene trees were used, and that MP-EST trees based on BestML gene trees generally produced more accurate species tree topologies for datasets with large numbers of genes (such as some of the model conditions studied in this paper). The explanation offered for this is that BestML gene trees are generally closer to the true gene tree than MLBS gene trees, and that this helps coalescent-based species tree estimation. Hence, the evaluation of the impact of binning on MP-EST with BestML gene trees is also needed. It is also possible that better results would be obtained using Bayesian methods (such as MrBayes [44]), rather than MLBS, to generate the distribution of gene trees [45], since the posterior distribution produced by Bayesian MCMC methods may be more closely centered around the true gene tree than the MLBS sample.

This study suggests that substantial improvement in species tree estimation could be obtained if we can develop more accurate methods for gene tree estimation. For example, methods that co-estimate gene sequence alignments and trees, such as BAli-Phy [46], SATé [47, 48], and PASTA [49], might provide improved gene tree estimation accuracy, compared to standard two-step procedures for estimating trees (first align, and then compute the tree).

Indeed, another challenge is that *if* loci are restricted to ultra-short sequences (10–50 sites), so as to decrease the probability of intra-locus recombination, then approaches based on combining estimated gene trees may not be able to provide highly accurate results, no matter what techniques are used to estimate gene trees. Hence, it is also possible that methods that construct species trees directly from the sequence data, rather than by combining gene trees, will have the best accuracy (see, for example, [50–52]), since they can avoid the analytical and empirical challenges caused by gene tree estimation error.

However, as observed in this and other studies [24, 42], concatenation often produces more accurate trees than even the best coalescent-based methods when the level of ILS is low enough. Therefore, an important question is whether a given biological dataset has a sufficiently high level of ILS that a coalescent-based analysis is needed. Conversely, coalescent-based methods that are not only more accurate than concatenation under conditions with high ILS, but also comparably accurate even under low levels of ILS, would be very helpful tools.

Finally, since statistical binning did reduce accuracy for some of the data we examined with small numbers of species and the very highest ILS levels, an important question that needs to be addressed is whether these very high ILS simulation conditions explored here and elsewhere represent realistic levels of ILS, or whether they represent extreme conditions that are unlikely to be observed in nature. Accurate estimations of ILS levels in biological data would enable the research community to direct its efforts to developing methods that would have the greatest utility in practice.

Overall, this study confirms the general finding in [23] that highly accurate coalescent-based species tree estimation is possible, and that statistical binning used with good coalescent-based methods can provide improved accuracy relative to concatenation under many conditions.

## Materials and Methods

### Proofs

Recall that under the GTR+MSC model, gene trees evolve within a species tree under the multi-species coalescent (MSC) model, and then sequences evolve down each gene tree under the General Time Reversible (GTR) model. The different gene trees are equipped with their own GTR model parameters, and so the tree topologies, 4 × 4 substitution matrices, and gene tree branch lengths can differ between the different genes.

The main results of this section are given in Theorem 2 and Theorem 3, where we prove that using weighted statistical binning in a phylogenomic pipeline is statistically consistent under the GTR+MSC model, but that replacing weighted statistical binning with unweighted statistical binning is *not* statistically consistent under the GTR+MSC model, respectively.

The statistical binning algorithm uses a heuristic to color the vertices, which we now describe. Since each gene is associated with a vertex, we will describe the heuristic in terms of what it does with genes. The algorithm has two stages. In the first stage, we use a heuristic to find a large clique in the incompatibility graph (i.e., a set of pairwise incompatible genes) and we assign each gene in the clique to a different bin. Then, in the greedy stage, genes are processed in turn (according to an order described below), and each gene is placed in the bin with the smallest number of genes with which it has no strongly supported conflicts (where strongly supported conflict between two genes means that there are branches, one for each of the estimated gene trees, that are incompatible, and both branches have support above *B*, where *B* is the user provided bootstrap threshold support value). If no such bin exists, a new bin is created and the gene is placed in this new bin. If there are two or more bins with the same smallest number of genes into which the new gene can be placed, the tie is broken randomly. Genes are processed based on a dynamic ordering, such that the next selected gene is always the one incompatible with the largest number of existing bins (breaking ties arbitrarily). Therefore,

Lemma 1: Let 𝒯 = {*t*_1_, *t*_2_, …, *t*_*p*_} be the multi-set of estimated gene trees for *p* genes *g*_1_, *g*_2_, …, *g*_*p*_, and assume that all the branches in each *t*_*i*_ have bootstrap support above *B*, the user-provided bootstrap support threshold. Then, when statistical binning is run, there will be one bin for each of the different estimated gene tree topologies in 𝒯, and for every bin, every two genes in the bin will have the same estimated gene tree topology.

Proof: Our inductive hypothesis is that after placing *K* genes into bins, there will be one bin for each of the estimated gene tree topologies for this set of *K* genes, and that every two genes in any bin will have the same estimated gene tree topology. We will prove the lemma true by induction on *K* ≥ 1.

For *K* = 1, it is trivially true. Now suppose the inductive hypothesis holds for *K* − 1 genes, and consider what happens when the *K* ^*th*^ gene, *g*_*K*_, is placed. Recall that the algorithm operates in two stages: first it finds a clique in the graph and places the genes within that clique into different bins, and then it enters the greedy phase. We can consider the genes within the clique to be arbitrarily ordered, and placed in bins using that order. There are two cases to consider, depending on whether *g*_*K*_ is part of the initial clique found by the algorithm. If *g*_*K*_ *is* part of the initial clique, then *g*_*K*_ is placed in a separate bin by itself, and has a different topology from the *K* − 1 genes that preceded it (because these are also in the initial clique, and are placed in bins by themselves). If *g*_*K*_ is *not* part of the initial clique, then it is placed in a bin during the greedy stage of the algorithm. By the inductive hypothesis, the algorithm has placed the first *K* − 1 genes into bins, there is a single bin for each of the different estimated gene tree topologies observed among the first *K* − 1 genes, and every two genes in any bin have the same estimated gene tree topology. When we process *g*_*K*_, there are two cases, depending on whether its estimated gene tree *t*_*K*_ is a gene tree topology that has been seen before. If *t*_*K*_ = *t*_*i*_ for some 1 ≤ *i* ≤ *K* − 1, then there is a bin that contains all the genes with that topology, and *g*_*K*_ can be added to that bin. Note that by the inductive hypothesis, all other bins contain genes with different estimated gene tree topologies than *t*_*K*_. Furthermore, by assumption, all edges of all gene trees have bootstrap support above *B*. Hence, we cannot add *g*_*K*_ to any other bin. Therefore, if *t*_*K*_ has been seen before, there is only one bin we can add *g*_*K*_ to, and it is the bin for genes with the same tree topology as *t*_*K*_. The other case is where *t*_*K*_ has not been seen before. In this case, *t*_*K*_ is different from every previously seen gene tree, and so a new bin is created. As a result, the new set of bins satisfies the inductive hypothesis, so that there is one bin for every estimated gene tree topology, and no two genes in any bin have different estimated gene tree topologies.

Therefore,

Theorem 1: Let *T* ^*sp*^ be a species tree with branch lengths in coalescent units, and 𝒯 = {*t*_1_, *t*_2_, …, *t*_*p*_} be a set of *p* rooted gene trees sampled from the distribution defined by *T* ^*sp*^ under the multi-species coalescent model. Let {*θ*_1_, *θ*_2_, …, *θ*_*p*_} be a set of numeric GTR model parameters (gene tree branch lengths and 4 × 4 substitution matrices) so that *T*_*i*_ = (*t*_*i*_, *θ*_*i*_) is a GTR model tree for each *i* = 1, 2, …, *p*. Let 𝒯′ = {*T*_1_, *T*_2_, …, *T*_*p*_}. For each *i*, 1 ≤ *i* ≤ *p*, let sequence dataset *S*_*i*_ evolve down the GTR model tree *T*_*i*_. Let *ϵ* < 1 and bootstrap support threshold *B* < 1 be given. Then, there is a sequence length *L* (that depends on 𝒯′ and *ϵ*) such that if at least *L* sites evolve down each gene tree, then with probability at least 1−*ϵ*, the following will be true:

- For each *i* = 1, 2, …, *p*, the gene tree estimated using GTR maximum likelihood on *S*_*i*_ will have the same unrooted topology as *t*_*i*_ (the true gene tree for *S*_*i*_), and will have bootstrap support greater than *B* for all its branches,
- For every bin produced by statistical binning based on GTR maximum likelihood analyses of the gene sequence alignments, the estimated gene trees for genes in the bin will have the same topology, and
- All genes with the same true gene tree topology will be in the same bin.

Proof: Since GTR maximum likelihood is statistically consistent for sequences generated by GTR model trees, then for any *ϵ*′ > 0, there is a sequence length *L*_*i*_ such that given sequence dataset *S*_*i*_ with at least *L*_*i*_ sites generated on *T*_*i*_, the GTR maximum likelihood tree topology for *S*_*i*_ is *t*_*i*_ (i.e., the true gene tree) and has bootstrap support greater than *B*, with probability at least 1−*ϵ*′. Letting *L* = max_*i*_{*L*_*i*_}, it follows that all estimated gene trees will be the true gene trees and have bootstrap support greater than *B* with probability at least 1−*pϵ*′. Therefore, when $\epsilon^{\prime }=\frac{\epsilon }{p}$ and the sequences are all of length at least *L*, the result then follows by Lemma 1.

Fully partitioned GTR maximum likelihood: In a fully partitioned GTR maximum likelihood analysis, the input is a set of *p* multiple sequence alignments, {*S*_1_, *S*_2_, …, *S*_*p*_}. These alignments are concatenated into a supermatrix, *M*, in which the locations where the different alignments begin and end are also noted. The maximum likelihood score of a candidate tree *t* (note that *t* specifies only a topology and not also branch lengths) for input *M* is

$$\begin{array}{c} score\left(t\right)=sup_{\Theta }\left\{\prod_{i}^{p}Pr\left(S_{i}|\left(t,\theta_{i}\right)\right):\Theta =\left\{\theta_{1},\theta_{2},\ldots ,\theta_{p}\right\}\right\} \end{array}$$ (1)

Thus, Θ denotes a set of GTR model parameters (branch lengths and GTR substitution matrix) for each of the parts within the concatenated alignment *M*. We will refer to the tree topology that achieves the optimal score under this fully partitioned analysis as the solution to the fully partitioned maximum likelihood analysis of the concatenated matrix, understanding that the numeric GTR parameters (branch lengths and substitution matrices) are estimated independently for each part of the alignment, and hence can differ arbitrarily between parts.

Lemma 2: Let *S* be a set of taxa, and let *S*_*i*_ be a set of DNA sequences for *S*, with *i* = 1, 2, …, *p*. Suppose that tree topology *t* is an optimal solution for GTR maximum likelihood for each *S*_*i*_ (allowing various GTR parameters for different *i* = 1, 2, …, *p*). Then *t* will be an optimal solution to a fully partitioned GTR maximum likelihood analysis on a concatenation of *S*_1_, *S*_2_, …, *S*_*p*_.

Proof: Recall that in a fully partitioned GTR maximum likelihood analysis, the maximum likelihood score of a given candidate tree *t* with respect to a matrix *M* under a fully partitioned ML analysis is given by Eq (1). Suppose that the tree topology *t* is an optimal solution to GTR maximum likelihood for each *S*_*i*_ but not an optimal solution to the fully concatenated GTR maximum likelihood analysis. Then, for some tree *t*′ ≠ *t*, *score*(*t*′) > *score*(*t*). Therefore, for at least one *i*, *sup*_*θ*_{*Pr*(*S*_*i*_|(*t*′, *θ*)} > *sup*_*θ*_{*Pr*(*S*_*i*_|(*t*, *θ*))}. But then *t* is not an optimal GTR maximum likelihood tree topology for *S*_*i*_, contradicting our assumption. Therefore, if the maximum likelihood analysis is performed in a fully partitioned manner, then tree topology *t* will be an optimal solution to the GTR maximum likelihood analysis.

Comments: The use of a fully partitioned analysis that enables different parameters for different partitions is critically important for the proof. Consider, for example, the result that would be obtained given a set of *p* sequence alignments for *n* species, of which *p*−1 of them are constant (meaning all the sequences are identical across all the species), but one sequence alignment, *S*_*p*_, is obtained by evolving sequences down a GTR gene tree, *T*. In a fully partitioned GTR maximum likelihood analysis, the *p*−1 multiple sequence alignments that exhibit no changes do not impact the solution to maximum likelihood, because they have the same score for every possible tree topology. Therefore, the outcome of a fully partitioned GTR maximum likelihood analysis of the concatenated alignment will simply have the GTR maximum likelihood tree topology for *S*_*p*_ (recall that a fully partitioned analyses does not produce one unique set of branch lengths or other model parameters). However, in an *unpartitioned* GTR maximum likelihood analysis, the result can be quite different—because the *p*−1 alignments without changes on them will drive down the estimated branch lengths, which are held in common across all the sites. See [6] for an analysis of the theoretical properties of unpartitioned maximum likelihood in the context of the multi-species coalescent model. We now consider the result of applying weighted statistical binning within a phylogenomic pipeline.

Corollary 1: Let 𝒢 = {*g*_1_, *g*_2_, …, *g*_*p*_} be a set of *p* genes, and *T*_*i*_ = (*t*_*i*_, *θ*_*i*_) be the true gene tree and GTR parameters (including branch length) for *g*_*i*_, *i* = 1, 2, …, *p*. Let *B* < 1 be the user provided bootstrap support value. Assume that the gene sequence alignment *S*_*i*_ evolves down the GTR model tree *T*_*i*_ = (*t*_*i*_, *θ*_*i*_), for *i* = 1, 2, …, *p*. As the sequence lengths for all the genes increase then with probability converging to 1, for each bin produced during a statistical binning analysis, the estimated gene trees will be the true gene trees, all genes in any bin will have the same estimated and true gene tree, and the supergene trees produced for each bin will converge in probability to the common true gene tree for the genes in the bin.

Proof: By Theorem 1, as the sequence length increases, then with probability converging to 1, the genes in each bin will share a common true gene tree topology, their estimated gene trees will be topologically identical to each other and to the true gene tree, and will each have bootstrap support greater than *B*. By Lemma 2, under these conditions, a fully partitioned GTR maximum likelihood analysis of the concatenated alignment of the genes in a bin will produce the true gene tree topology for the genes in the bin.

We now address the statistical consistency of phylogenomic pipelines that use weighted and unweighted statistical binning.

Theorem 2: The phylogenomic pipeline that uses GTR maximum likelihood to estimate gene trees, uses weighted statistical binning to compute supergene trees, and then combines the supergene trees using a coalescent-based summary method, is statistically consistent under the GTR+MSC model.

Proof: We begin with the proof of statistical consistency for weighted statistical binning. By Corollary 1, as the sequence length for each gene goes to infinity (*k* → ∞) all genes put in any bin by statistical binning will have the same true gene tree with probability converging to 1, and the supergene trees produced for each bin will converge in probability to this common true gene tree. In weighted statistical binning, this common true gene tree topology is replicated as many times as the number of genes in the bin, and hence the distribution produced using weighted statistical binning is identical to the distribution of the unbinned true gene trees. Therefore, as both *k* and *p* increase, the gene tree distribution produced by weighted statistical binning converges to the true gene tree distribution. The statistical consistency of the pipeline follows from the use of a coalescent-based summary method, since as *p* → ∞, the species tree produced by the summary method given true gene trees converges to the true species tree.

We now consider the case where we use unweighted statistical binning instead of weighted statistical binning.

Theorem 3: The phylogenomic pipeline that uses GTR maximum likelihood to estimate gene trees, uses unweighted statistical binning to compute supergene trees, and then combines the supergene trees using a coalescent-based summary method, is statistically inconsistent under the GTR+MSC model.

Proof: The proof for Theorem 2 shows that as the sequence length *k* increases, the set of bins produced by statistical binning converges in probability to having one bin for each of the true gene trees, and the supergene tree for each bin converges to the common true gene tree for the bin. As *p* → ∞, the set 𝒯 converges in probability to the set of all possible gene trees (since all gene trees have strictly positive probability under the multi-species coalescent model). Hence, the multi-set of supergene trees produced by unweighted statistical binning will converge to the set that has each possible gene tree appearing exactly once. This is a flat distribution, and it is not possible to reconstruct the species tree from a flat distribution. Hence, the use of unweighted statistical binning in a phylogenomic pipeline is not statistically consistent.

### Evaluation

We explored the performance of MP-EST and ASTRAL with weighted and unweighted statistical binning, and also without binning. We also examine concatenation of the entire set of gene sequence alignments using an unpartitioned maximum likelihood analysis using RAxML. We explore performance on a collection of simulated and biological datasets originally studied in [23]. We applied MP-EST and ASTRAL to a set of RAxML gene trees computed on bootstrap replicates of each gene sequence alignment. With bootstrap ML gene trees for each gene, summary methods were applied with the site-only multi-locus bootstrapping (MLBS) procedure [53], implemented as follows. For each gene or supergene, 200 replicates of bootstrapping are performed using RAxML. Next, 200 replicates (*R*_1_, *R*_2_, …, *R*_200_) of input datasets to the summary methods are created such that *R*_*i*_ contains the *i* ^*th*^ bootstrap tree across all genes/supergenes. The summary methods are then run on these 200 input replicates, and 200 species trees are estimated. Finally, the greedy consensus tree of these 200 estimated species tree is computed, and support values are drawn on the branches of the greedy consensus tree by counting the occurrences of each bipartition in the 200 species trees.

#### Triplet gene tree distribution error

MP-EST computes species trees using the estimated distribution on rooted triplet trees defined by its input of gene trees. We therefore evaluated the impact of binning on the estimated gene tree distribution, measuring the divergence between the triplet distribution of estimated gene trees and the triplet distribution of true gene trees. We represent the gene tree distribution by the frequency of each of the three possible alternative topologies for all the $\left(\begin{array}{c} n\\ 3 \end{array}\right)$ triplets of taxa, where *n* is the number of taxa. Therefore, we have $\left(\begin{array}{c} n\\ 3 \end{array}\right)$ true triplet distributions. Hence, for each triplet of taxa, we have estimated triplet distributions using the unbinned analysis, as well as weighted and unweighted binning analyses. We computed the Jensen-Shannon divergence of each of these $\left(\begin{array}{c} n\\ 3 \end{array}\right)$ triplet distributions and showed the empirical cumulative distribution of these divergences. The Jensen-Shanon divergence is a symmetrized and smoothed version of Kullback-Leibler divergence [54] between two distributions *P* and *Q*, and can be calculated as follows [55]:

$$\begin{array}{c} JS\left(P,Q\right)=\frac{1}{2}KL\left(P,M\right)+\frac{1}{2}KL\left(Q,M\right) \end{array}$$ (2)

where $M=\frac{P+Q}{2}$, and KL is the Kullback-Leibler divergence.

#### Species tree estimation error and branch support

We compared the estimated species trees to the model (i.e., true) species tree (for the simulated datasets) or to the scientific literature (for the biological datasets). We measure topological error using the missing branch rate (also known as the false negative (FN) rate), which is the proportion of branches in the true tree that are missing from the estimated tree. We also reported the error in species tree branch lengths estimated by MP-EST using the ratio of estimated branch length to true branch length for those branches of the true tree that appear in the estimated tree; thus, 1 indicates correct estimation, values above 1 indicate lengths that are too long, and values below 1 indicate branch lengths that are too short. Note that species tree branch lengths reflect the expected amount of ILS, and so under-estimation of species tree branch lengths means over-estimation of ILS, and over-estimation of branch lengths means under-estimation of ILS. We also computed the branch support of the false positive (FP) and true positive (TP) edges, where false positive edges are present in the estimated tree but not in the true tree, and edges that are present in both the estimated and true tree are true positive edges.

### Simulated datasets

We studied four collections of simulated datasets: two based on biological datasets that were generated in a prior study [23], and two new collections with smaller numbers of species. We briefly describe the simulation protocol for the biological datasets, and direct the reader to [23] for full details.

#### Mammalian simulated datasets

This dataset was generated by [23], and studied there and also in [43]. Here we describe the procedure followed by [23] to generate these data. First, a species tree was computed for the full biological dataset in [31], using MP-EST (this was done before removing 23 erroneous genes), and the tree topology and branch lengths were used as the model tree. Thus, the mammalian simulation model tree has an ILS level based on an MP-EST analysis of the biological mammalian dataset. Gene trees were simulated within this species tree under the multi-species coalescent model, and then the branch lengths on the gene trees were defined using the gene trees estimated on the biological dataset.

Variants of the basic model condition were generated by varying the amount of ILS, the number of genes, and the sequence length for each gene; these modifications also impact the amount of gene tree estimation error and the average bootstrap support in the estimated gene trees, and so can be modified to produce datasets that resemble the biological data.

The amount of ILS was varied by adjusting the branch length (shorter branches increase ILS). A model condition with reduced ILS was created by uniformly doubling (2X) the branch lengths, and a model condition with higher ILS was generated by uniformly dividing the branch lengths by two (0.5X). The amount of ILS obtained without adjusting the branch lengths is referred to as “default ILS”, and was estimated by MP-EST on the biological data.

The average bootstrap support (BS) in the biological data was 71%, and so [23] generated sequence lengths that produced estimated gene trees with bootstrap support bracketing that value—500bp alignments produced estimated gene trees with 63% average BS and 1000bp alignments produced estimated gene trees with 79% BS. We also generated model conditions with very short sequence lengths (250bp), which have 43% average BS.

Here, we varied the number of genes from 50 to 800 to explore both smaller and larger numbers of genes than the biological dataset (which had roughly 400 genes). In total, we generated 17 different model conditions specified by the ILS level, the number of genes, and the sequence length. For each of these model conditions, [23] created 20 replicates.

#### Avian simulated datasets

Mirarab et al. [23] used the species tree estimated by MP-EST on a subset of the avian dataset with 48 species and 14,446 loci studied by [26], and simulated gene trees by varying different parameters (similar to the mammalian simulated datasets). Three types of genomic markers were studied in [26]: exons, UCEs, and introns. The average bootstrap support (BS) of the gene trees based on exons, UCEs, and introns, was 24%, 39% and 48%, respectively; the longest introns had the highest average BS (59%). Mirarab et al. varied sequence lengths (250bp, 500bp, 1000bp, and 1500bp) to produce four model conditions with patterns of average bootstrap support that resemble these four marker types. Mirarab et al. varied the number of genes from 200 to 2000, but here, we augmented the dataset to also look at fewer genes (50 and 100). Mirarab et al. varied the amount of ILS, using the same technique as was used in generating the mammalian simulated datasets.

#### 15-taxon simulated datasets

We simulated a collection of 15-taxon datasets. The model species tree is a caterpillar-like ultrametric tree (i.e., the substitution process obeys a strict molecular clock) with 15 taxa; hence, it has two leaves *x* and *y* that are siblings in the tree. The lengths of all internal branches and the two branches incident with leaves *x* and *y* are all set to 0.005 substitutions per site; note that the assumption of ultrametricity defines the remaining branch lengths. The population size parameter (*θ* = 4*Nμ*) is set to 0.05 for all branches, and this results in 12 short internal branches (0.1 in coalescence units) in succession. Ultrametric gene trees were simulated down this tree using McCoal [56] and commands given in S13 Fig. Sequence data were simulated down each gene tree using bppseqgen [57] according to GTR+Γ parameters given in S13 Fig. We built four model conditions (with ten replicates each) by trimming gene data to 100 or 1000 sites and by exploring 100 or 1000 genes.

#### 10-taxon simulated datasets

We used simPhy [33] to simulate species trees using the Yule process with two different maximum tree length settings: 200K generations, resulting in short trees and high levels of ILS, and 1.8M generations, resulting in relatively longer trees and lower levels of ILS. We generated 20 species trees per model condition, and used simPhy to simulate 200 gene trees for each species trees using the multi-species coalescent process (simPhy parameters and commands are given in S14 Fig). The gene trees (with branch lengths in substitution units) deviate from the strict molecular clock, and the rates of evolution vary across genes. We used Indelible to simulate GTR+Γ sequence evolution down these gene trees with 100 sites, with parameters given in S14 Fig.

### Biological datasets

We studied two biological datasets also studied in [23]: the avian dataset [26] containing 14,446 loci across 48 species, and a reduced version of the mammalian dataset studied by Song et al. [31] with 447 loci across 37 species, from which [23] deleted 23 erroneous genes and re-estimated gene trees using RAxML (see [23, 43] for discussion of these loci).

### Methods and commands

#### Gene tree estimation

RAxML version 7.3.5 [58] was used to estimate gene trees under the GTRGAMMA model, using the following command:

raxmlHPC-SSE3 -m GTRGAMMA -s [input_alignment] -n [output_name] -N 20 -p [random_seed_number]

The following command was used for bootstrapping:

raxmlHPC-SSE3 -m GTRGAMMA -s [input_alignment] -n [output_name] -N 200 -p [random_seed_number] -b [random_seed_number]

#### Supergene tree estimation

For the biological datasets and the 10- and 15-taxon simulated datasets, we used a fully partitioned maximum likelihood analysis. All other analyses were based on unpartitioned maximum likelihood analysis, using the command given above for gene tree estimation. For the fully partitioned analysis, we used the following command:

raxmlHPC-SSE3 -m GTRGAMMA -s [input_alignment] -m GTRGAMMA -n [output_name] -N 20 -M -q [partition_file] -p [random_seed_number]

#### Concatenation

We concatenate the alignments of all genes into one supermatrix, and then estimate a tree from the supermatrix using unpartitioned maximum likelihood. We computed a parsimony starting tree using RAxML version 7.3.5, and then ran RAxML-light version 1.0.6. The following commands were used:

raxmlHPC-SSE3 -y -s supermatrix.phylip -m GTRGAMMA -n [output_name] -p [random_seed_number]
raxmlLight-PTHREADS -T 4 -s supermatrix.phylip -m GTRGAMMA -n name -t [parsimony_tree]

#### MP-EST

We used version 1.3 of MP-EST. We ran MP-EST 10 times with different random seed numbers, and selected the species tree with the best likelihood score using a custom shell script. MP-EST was run using site-only multi-locus bootstrapping, using 200 MLBS replicates, and returning the greedy consensus of the 200 MP-EST MLBS species trees as the output. The branch support on the edges of the tree represent the frequency of the bipartition in the sample of 200 species trees.

#### ASTRAL

We used ASTRAL version 4.7.6. in its default mode using the following command:

astral.4.7.6.jar -i [input_gene_trees] -o [output_file]

#### Greedy consensus

The greedy consensus (also called the “extended majority consensus”) of a set of trees, all on the same set of leaves, is obtained by ordering the bipartitions that appear in one or more trees in the order of their frequency (most frequent first). Then, a tree is built from this set, beginning with the first bipartition, and then modifying the tree to include the next bipartition in the list, if the addition of the bipartition is possible. We used Dendropy version 3.12.0 [59] to compute greedy consensus trees when running MP-EST or ASTRAL with MLBS gene trees.

### Data Availability

Most of the datasets used in this study are available through the prior publications. The new datasets generated for this study are available on figshare, with DOI: http://dx.doi.org/10.6084/m9.figshare.1411146. (Retrieved May 13, 2015.) The weighted statistical binning software is available on github at https://github.com/smirarab/binning (Retrieved May 14, 2015.)

## Supporting Information

S1 Table Gene tree estimation error, with and without binning for simulated datasets. We show the average gene tree estimation error for the simulated datasets analyzed in this paper. Results are shown for fixed number of genes (1000 for avian and 200 for mammalian, 100 for 15-taxon and 100 for 10-taxon). We fixed the level of ILS to 1X for avian, mammalian and 15-taxon datasets; and varied the level of ILS for 10-taxon datasets with 100bp sequence length. Gene tree error is mean topological distance, measured using the missing branch rate between the true gene tree and all 200 bootstrap replicates of each estimated gene tree. For the supergene trees, each bootstrap replicate of each supergene tree is compared separately against each true gene tree for the genes put in that bin. “n.a.” stands for “not available”.
(PDF)

S2 Table Average bootstrap support of estimated gene trees. We show the average bootstrap support values of the estimated gene trees for the simulated datasets. Results are shown for fixed number of genes (1000 for avian and 200 for mammalian, 100 for 15-taxon and 100 for 10-taxon datasets). We fixed the level of ILS to 1X for avian, mammalian and 15-taxon datasets; and varied the level of ILS for 10-taxon datasets with 100bp sequence length.
(PDF)

S1 Fig Effect of binning on the branch lengths (in coalescent units) estimated by MP-EST using MLBS on the avian simulated datasets with varying numbers of gene trees. We show the species tree branch length error (the ratio of estimated branch length to true branch length for branches of the true tree that appear in the estimated tree; 1 indicates correct estimation). We varied the number of genes from 50 to 2000, and fixed the sequence length to 500bp with default amount of ILS (1X level). We used 50% bootstrap support threshold for binning. Supergene trees were estimated using unpartitioned analyses.
(EPS)

S2 Fig Cumulative distribution of the bootstrap support values (obtained using MLBS) of true positive (TP) and false positive (FP) edges estimated by MP-EST on avian datasets. We varied the numbers of genes, and fixed the sequence length to 500bp (UCE-like) with default amount of ILS (1X level). We used 50% bootstrap support threshold for binning. Supergene trees were estimated using unpartitioned analyses. To produce the graph, we order the branches in the estimated species tree by their quality, so that the true positives with high support come first, followed by lower support true positives, then by false positives with low support, and finally by false positives with high support. The false positive branches with support above 75% are the most troublesome, and that fraction are indicated in the grey area. When the curve for a method lies above the curve for another method, then the first method has better bootstrap support.
(EPS)

S3 FigDivergence of estimated gene trees triplet distributions from true gene tree distributions for simulated mammalian datasets.(a) Varying gene sequence alignments lengths with 200 number of genes and default levels of ILS (1X); (b) varying ILS levels with fixed 200 genes and sequence length fixed to 500bp (63% BS). We used 75% bootstrap support threshold for binning. Supergene trees were estimated using unpartitioned analyses. True triplet frequencies are estimated based on true gene trees for each of the $\left(\begin{array}{c} n\\ 3 \end{array}\right)$ possible triplets, where *n* is the number of species. Similarly, triplet frequencies are calculated from estimated gene/supergene trees. For each of these $\left(\begin{array}{c} n\\ 3 \end{array}\right)$ triplets, we calculate the Jensen-Shannon divergence of the estimated triplet distribution from the true gene tree triplet distribution. We show the empirical cumulative distribution of these divergences. The empirical cumulative distribution shows that for a given divergence level, what percentage of the triplets are diverged from true triplet distribution at or below that level. Results are shown for 10 replicates.(EPS)

S4 Fig Species tree estimation error for MP-EST with MLBS on mammalian simulated datasets with varying amounts of ILS. We show average FN rate over 20 replicates. We varied the amount of ILS, and fixed the number of genes to 200 and sequence length to 500bp (63% BS). We used 75% bootstrap support threshold for binning. Supergene trees were estimated using unpartitioned analyses.
(EPS)

S5 Fig Effect of binning on the branch lengths (in coalescent units) estimated by MP-EST using MLBS on the mammalian simulated datasets with varying amounts of ILS. We show the species tree branch length error (the ratio of estimated branch length to true branch length for branches of the true tree that appear in the estimated tree; 1 indicates correct estimation). We varied the amount of ILS, and fixed the number of genes to 200 and sequence length to 500bp (63% BS). We used 75% bootstrap support threshold for binning. Supergene trees were estimated using unpartitioned analyses.
(EPS)

S6 Fig Cumulative distribution of the bootstrap support values (obtained using MLBS) of true positive (TP) and false positive (FP) edges estimated by MP-EST on mammalian datasets. We varied the numbers of genes, and gene sequence alignments length with default amount of ILS. We used 75% bootstrap support threshold for binning. Supergene trees were estimated using unpartitioned analyses. To produce the graph, we order the branches in the estimated species tree by their quality, so that the true positives with high support come first, followed by lower support true positives, then by false positives with low support, and finally by false positives with high support. When the curve for a method lies above the curve for another method, then the first method has better bootstrap support.
(EPS)

S7 Fig Cumulative distribution of the bootstrap support values (obtained using MLBS) of true positive (TP) and false positive (FP) edges estimated by MP-EST on mammalian datasets with varying amounts of ILS. We varied the amount of ILS, and fixed the number of genes to 200 and sequence length to 500bp. We used 75% bootstrap support threshold for binning. Supergene trees were estimated using unpartitioned analyses. To produce the graph, we order the branches in the estimated species tree by their quality, so that the true positives with high support come first, followed by lower support true positives, then by false positives with low support, and finally by false positives with high support. When the curve for a method lies above the curve for another method, then the first method has better bootstrap support.
(EPS)

S8 Fig Species trees estimated by unbinned ASTRAL using MLBS on avian biological datasets. Branches without designation have 100% support. We used 50% bootstrap support threshold for binning. Supergene trees were estimated using fully partitioned analyses.
(EPS)

S9 Fig Species trees estimated by binned (with and without weighting) ASTRAL using MLBS on avian biological datasets. (a) Unweighted binned ASTRAL, and (b) weighted binned ASTRAL. Branches without designation have 100% support. We used 50% bootstrap support threshold for binning. Supergene trees were estimated using fully partitioned analyses. Binned ASTRAL with weighting and binned ASTRAL without weighting differ only in the placement of *Opisthocomus hoazin*. However, the branches supporting different placements of *Opisthocomus hoazin* have low support values (47% for unweighted binning and 55% for weighted binning).
(EPS)

S10 Fig Species trees estimated by unbinned MP-EST using MLBS for mammalian biological datasets. Branches without designation have 100% support. We used 75% bootstrap support threshold for binning. We estimated the supergene trees using fully partitioned analyses.
(EPS)

S11 Fig Species trees estimated by binned (with and without weighting) MP-EST using MLBS for mammalian biological datasets. Binned and unbinned ASTRAL returned identical topology. The branches on this tree are labeled with two support values side by side: the first one corresponds to unweighted binning and the next one corresponds to weighted binning. Branches without designation have 100% support. We used 75% bootstrap support threshold for binning. Supergene trees were estimated using fully partitioned analyses.
(EPS)

S12 Fig Species trees estimated by unbinned and binned (with and without weighting) ASTRAL using MLBS for mammalian biological datasets. Binned and unbinned ASTRAL returned identical topology. The branches on this tree are labeled with three support values side by side: the first one corresponds to unbinned ASTRAL, the next one corresponds to unweighted binning, and the last one is for weighted binning. Branches without designation have 100% support. We used 75% bootstrap support threshold for binning. Supergene trees were estimated using fully partitioned analyses.
(EPS)

S13 Fig Simulation parameters and commands for the 15-taxon datasets. (PDF)

S14 Fig Simulation parameters and commands for the 10-taxon datasets. (PDF)
