# Supplementary material for: Weighted Statistical Binning: Enabling Statistically Consistent Genome-Scale Phylogenetic Analyses
Source: PLoS One. 2015 Jun 18;10(6):e0129183. doi: 10.1371/journal.pone.0129183 (PMC4472720; doi:10.1371/journal.pone.0129183)
Supplement: S1 Table — We show the average gene tree estimation error for the simulated datasets analyzed in this paper. Results are shown for fixed number of genes (1000 for avian and 200 for mammalian, 100 for 15-taxon and 100 for 10-taxon). We fixed the level of ILS to 1X for avian, mammalian and 15-taxon datasets; and varied the level of ILS for 10-taxon datasets with 100bp sequence length. Gene tree error is mean topological distance, measured using the missing branch rate between the true gene tree and all 200 bootstrap replicates of each estimated gene tree. For the supergene trees, each bootstrap replicate of each supergene tree is compared separately against each true gene tree for the genes put in that bin. “n.a.” stands for “not available”. (PDF) [file pone.0129183.s001.pdf]

Dataset	Model condition	Gene tree error (%)		
		Unbinned	Binned-50	Binned-75
Avian	250bp	79	57	n.a.
	500bp	69	57	n.a.
	1000bp	55	51	n.a.
	1500bp	46	45	n.a.
Mammalian	250bp	60	n.a.	47
	500bp	43	n.a.	35
	1000bp	27	n.a.	26
15-taxon	100bp	77	80	86
	1000bp	36	36	40
10-taxon	Lower ILS	64	58	51
	Higher ILS	69	73	80

Table S1. Gene tree estimation error, with and without binning for simulated datasets. We show the average gene tree estimation error for the simulated datasets analyzed in this paper. Results are shown for fixed number of genes (1000 for avian and 200 for mammalian, 100 for 15-taxon and 100 for 10-taxon). We fixed the level of ILS to 1X for avian, mammalian and 15-taxon datasets; and varied the level of ILS for 10-taxon datasets with 100bp sequence length. Gene tree error is mean topological distance, measured using the missing branch rate between the true gene tree and all 200 bootstrap replicates of each estimated gene tree. For the supergene trees, each bootstrap replicate of each supergene tree is compared separately against each true gene tree for the genes put in that bin. “n.a.” stands for “not available”.
