# Supplementary material for: Weighted Statistical Binning: Enabling Statistically Consistent Genome-Scale Phylogenetic Analyses
Source: PLoS One. 2015 Jun 18;10(6):e0129183. doi: 10.1371/journal.pone.0129183 (PMC4472720; doi:10.1371/journal.pone.0129183)
Supplement: S2 Table — We show the average bootstrap support values of the estimated gene trees for the simulated datasets. Results are shown for fixed number of genes (1000 for avian and 200 for mammalian, 100 for 15-taxon and 100 for 10-taxon datasets). We fixed the level of ILS to 1X for avian, mammalian and 15-taxon datasets; and varied the level of ILS for 10-taxon datasets with 100bp sequence length. (PDF) [file pone.0129183.s002.pdf]

Dataset	Model condition	Average bootstrap support (%)
Avian	250bp	27
	500bp	31
	100bp	51
	1500bp	60
Mammalian	250bp	43
	500bp	63
	1000bp	79
15-taxon	100bp	35
15-taxon	1000bp	70
10-taxon	Lower ILS, 100bp	45
	Higher ILS, 100bp	37

Table S2. Average bootstrap support. We show the average bootstrap support values of the estimated gene trees for the simulated datasets. Results are shown for fixed number of genes (1000 for avian and 200 for mammalian, 100 for 15-taxon and 100 for 10-taxon datasets). We fixed the level of ILS to 1X for avian, mammalian and 15-taxon datasets; and varied the level of ILS for 10-taxon datasets with 100bp sequence length.
