# Supplementary material for: Weighted Statistical Binning: Enabling Statistically Consistent Genome-Scale Phylogenetic Analyses
Source: PLoS One. 2015 Jun 18;10(6):e0129183. doi: 10.1371/journal.pone.0129183 (PMC4472720; doi:10.1371/journal.pone.0129183)
Supplement: S13 Fig — (PDF) [file pone.0129183.s015.pdf]

a) Control files used for MCcoal simulations (MCcoal.ctl):

```
SimulatedData.txt
9823126266
15 A B C D E F G H I J K L M N O
  1 1 1 1 1 1 1 1 1 1 1 1 1 1 1
((((((((((((((A #.05,B #.05):0.005 #.05,C #.05):0.01 #.05, D #.05):0.015
#.05,E #.05):0.02 #.05,F #.05):0.025 #.05,G #.05):0.03 #.05,H #.05):0.035
#.05,I #.05):0.04 #.05,J #.05):0.045 #.05,K #.05):0.05 #.05,L #.05):0.055
#.05,M #.05):0.06 #.05,N #.05):0.065 #.05,O #.05):0.565 #.05;
```

b) Command used to run MCcoal

```
   printf "10000 1000" PATH_TO_MCCOAL/MCcoal
```

c) Commands to run bppseqgen

```
mkdir allTrees;
split -a 4 -l 1 out.trees;
for i in x* ; do mv $i  allTrees/; done
for i in allTrees/x* ; do
 bppseqgen number_of_sites=1000 input.tree.file=$i param=opts output.sequence.file=$i".fasta"
done
```

d) GTR+Γ model parameters

```
model = GTR(a=1.062409952497, b=0.133307705766, c=0.195517800882,
d=0.223514845018, e=0.294405416545,
theta=0.469075709819, theta1=0.558949940165, theta2=0.488093447144)
rate_distribution = Gamma(n=4, alpha=0.370209777709)
```

Figure S13. Simulation parameters and commands for the 15-taxon datasets. Gene trees were simulated using MCcoal, with control files given here (a) and the command provided (b). The control files define the species tree, which is in the caterpillar form. Running MCcoal simulated 10,000 gene trees, which we divided into 10 replicates of 1000 genes or 100 genes. For each true gene tree, we then simulated alignments using bppseqgen, using the command given in (c). Here, the file “opts” is the same file we used in [23] and defines parameters given in (d).
