# Supplementary material for: Weighted Statistical Binning: Enabling Statistically Consistent Genome-Scale Phylogenetic Analyses
Source: PLoS One. 2015 Jun 18;10(6):e0129183. doi: 10.1371/journal.pone.0129183 (PMC4472720; doi:10.1371/journal.pone.0129183)
Supplement: S14 Fig — (PDF) [file pone.0129183.s016.pdf]

a) Command used for SimPhy simulations:

```
for t in 1800000 200000; do
  b=0$(echo "scale=9; 1 / $t / 5"|bc -l}
  simphy -RS 20 -RL U:1000,1000 -RG 1 -ST U:$t,$t -SB U:$b,$b \\
            -SI U:1,1 -SL U:10,10 -CP U:400000,400000 -HS L:1.5,1 -HL L:1.2,1\\
            -HG L:1.4,1 -CU E:10000000 -SO U:1,1 -OD 1 -OR 0 -V 3  -CS 293745\\
            -O model.10.$t.$b |grep -E "[:-]"| tee log.10.$t.$b;
  for r in `ls -d model.$sp.$t.$b/*`; do
     sed -i -e "s/_0_0//g" $r/g_trees*.trees;
  done
done
```

b) Parameter settings for SimPhy:

Arg.	Description	Value	Notes
ST	maximum tree length	200K or 1.8M	
SB	birth rates	0.000001 or 0.000000111	
SI	number of individuals per species	1	
SL	number of leaves	10	
P	global population sizes	400000	
HS	Species-specific branch rate heterogeneity modifiers	Log normal (1.5,1)	
HL	Locus-specific rate heterogeneity modifiers	Log normal (1.2,1)	
HG	Gene-tree-branch-specific rate heterogeneity modifiers	Log normal (1.4,1)	
U	Global substitution rate	Exponential (10000000)	
SO	Outgroup branch length relative to half the tree length	1	

c) Indelible GTR+Γ model parameters

Base frequencies $\sim Dirichlet(36, 26, 28, 32)$
GTR Matrix $\sim Dirichlet(16, 3, 5, 5, 6, 15)$
Rate parameter $(\alpha) \sim Exponential(1.2)$ trimmed at 0.1 from below

Figure S14. Simulation parameters and commands for 10-taxon dataset. Gene trees were simulated using SimPhy with commands given here (a) which includes all the parameter settings (important parameters are listed in panel (b)). The maximum tree length parameter is set to either 1.8M or 200K to produce two different model conditions. The speciation rate parameter is adjusted based on the maximum tree length so that maximum rate multiplied by speciation rate is always 0.2 (thus, rate is 0.000000111 and 0.000001 for 1.8M and 200K respectively). We used a perl script available at `http://www.cs.utexas.edu/users/phylo/datasets/weighted-binning-datasets.html` to draw parameters for the Indelible simulations. Gene length is set to 1000 for all genes, but sequences are trimmed to their first 100bp in this study. For GTR+Γ parameters, we use a set of hyper parameters (estimated from real datasets) to drawn different parameter values for each gene in each replicate. Hyper parameters for base frequencies, GTR matrix, and the rate parameter (α) are shown in panel (c). These hyperparameters were calculated using maximum likelihood estimation form a collection of three large scale multi-locus datasets: 1KP dataset [35], Song et al. Mammalian dataset [31], and the Avian phylogenomics dataset [26]. The base values used for this maximum likelihood estimation and the corresponding scripts are available at `http://www.cs.utexas.edu/users/phylo/software/astral/` (under the first bullet; i.e., estimate-parameters.r). Note that for the shape frequency, α, we use a heavy-tailed distribution, but to avoid unrealistic settings, values below 0.1 are discarded.
